# APOE in the bullseye of neurodegenerative diseases: impact of the APOE genotype in Alzheimer’s disease pathology and brain diseases

**DOI:** 10.1186/s13024-022-00566-4

**Published:** 2022-09-24

**Authors:** Rosalía Fernández-Calle, Sabine C. Konings, Javier Frontiñán-Rubio, Juan García-Revilla, Lluís Camprubí-Ferrer, Martina Svensson, Isak Martinson, Antonio Boza-Serrano, José Luís Venero, Henrietta M. Nielsen, Gunnar K. Gouras, Tomas Deierborg

**Affiliations:** 1 Department of Experimental Medical Science, Experimental Neuroinflammation Laboratory, Lund University, Lund, Sweden; 2 Department of Experimental Medical Science, Experimental Dementia Research Unit, Lund University, Lund, Sweden; 3 Oxidative Stress and Neurodegeneration Group, Faculty of Medicine, Universidad de Castilla-La Mancha, Ciudad Real, Spain; 4 Departamento de Bioquímica Y Biología Molecular, Facultad de Farmacia, Universidad de Sevilla, and Instituto de Biomedicina de Sevilla-Hospital Universitario Virgen del Rocío/CSIC/Universidad de Sevilla, Seville, Spain; 5 Department of Biochemistry and Biophysics at, Stockholm University, Stockholm, Sweden

**Keywords:** Apolipoprotein E, Neuroinflammation, Alzheimer’s disease, Neurodegeneration

## Abstract

ApoE is the major lipid and cholesterol carrier in the CNS. There are three major human polymorphisms, apoE2, apoE3, and apoE4, and the genetic expression of *APOE4* is one of the most influential risk factors for the development of late-onset Alzheimer's disease (AD). Neuroinflammation has become the third hallmark of AD, together with Amyloid-β plaques and neurofibrillary tangles of hyperphosphorylated aggregated tau protein. This review aims to broadly and extensively describe the differential aspects concerning apoE. Starting from the evolution of apoE to how *APOE's* single-nucleotide polymorphisms affect its structure, function, and involvement during health and disease. This review reflects on how *APOE's* polymorphisms impact critical aspects of AD pathology, such as the neuroinflammatory response, particularly the effect of APOE on astrocytic and microglial function and microglial dynamics, synaptic function, amyloid-β load, tau pathology, autophagy, and cell–cell communication. We discuss influential factors affecting AD pathology combined with the *APOE* genotype, such as sex, age, diet, physical exercise, current therapies and clinical trials in the AD field. The impact of the *APOE* genotype in other neurodegenerative diseases characterized by overt inflammation, e.g., alpha- synucleinopathies and Parkinson's disease, traumatic brain injury, stroke, amyotrophic lateral sclerosis, and multiple sclerosis, is also addressed. Therefore, this review gathers the most relevant findings related to the *APOE* genotype up to date and its implications on AD and CNS pathologies to provide a deeper understanding of the knowledge in the *APOE* field.

## Background

Alzheimer’s disease (AD) is a progressive neurodegenerative disease characterized by gradual cognitive decline and memory loss. AD represents 60 to 70% of dementia cases and affects more than 50 million people worldwide [1]. Reported deaths from AD increased 148% between 2000 and 2018, and the economic burden worldwide was estimated at $305 billion for 2020 [1, 2].

The two main pathological hallmarks of AD are the accumulation of extracellular amyloid plaques and intraneuronal deposition of hyperphosphorylated microtubule-associated protein tau in the form of neurofibrillary tangles (NFTs). The classical hypothesis regarding AD’s pathophysiology is the amyloid cascade hypothesis postulated by Hardy and Higgins in 1992 [3]. Its premise is that amyloid plaques result from an imbalance between the production and clearance of amyloid-β (Aβ) peptides and that accumulation of Aβ peptides is the leading cause of AD. Aβ peptides are generated by the cleavage of the amyloid precursor protein (APP), a single-pass transmembrane- type-1 integral glycoprotein that can be cleaved and metabolized by α-secretases (non-amyloidogenic pathway) or by β-secretases (amyloidogenic pathway). Under physiological conditions, the Aβ peptide balance between production and clearance is maintained [4].

In the amyloidogenic pathway, APP is cleaved by β-secretase (also known as BACE-1), producing soluble APPβ (sAPPβ) and membrane-bound C-terminal 99 or 89 amino acid fragments, also known as β-CTF. Cleavage of APP CTFs leads to the extracellular secretion of Aβ peptides mainly composed of 39 to 42 amino acid length residues, in which Aβ_1-40_ and Aβ_1-42_ are predominant in vivo species. When soluble Aβ is over-produced, or its clearance is impaired, it self-assembles to form the amyloid fibrils constituting amyloid plaques. At certain levels and conformations, Aβ becomes toxic for neurons in many ways, inducing inflammation, oxidative stress, alterations in calcium homeostasis and membrane potentials, and cytoskeleton disruption [5–7]. Thus, according to the amyloid cascade hypothesis, Aβ accumulation also drives tau pathology. The physiological role of tau is closely related to the maintenance of neuronal morphology, synaptic plasticity, and axonal transport of organelles by its interaction with α-tubulin and β-tubulin [8–10]. Tau is encoded by the MAPT gene. There are six tau isoforms described in mammals, which differ in their microtubule-binding domains. Hyperphosphorylation of tau will negatively affect its binding properties to microtubules and enhance tau self-assembling capacity to form oligomers, fibrils, and eventually NFTs [11].

Multiple findings have called the Amyloid hypothesis into question in the past years. Some of the reasons are the failure of most anti-amyloid-based therapeutics, lack of neuropathological correlations between amyloid plaque density and disease severity, or lack of clinically predictive AD mouse models. Accordingly, new etiological hypotheses and strategies have grown, for instance, a focus on the senescence-associated secretory phenotype in the central nervous system (CNS) cells emerged as a promising therapeutic strategy for multiple age-related conditions [12, 13]. The prion-like propagation hypothesis was proposed for AD, Parkinson’s disease (PD), and Amyotrophic Lateral Sclerosis (ALS) based on the ability of misfolded proteins from neurodegenerative diseases to seed and propagate pathology in a prion-like manner [14]. The cholinergic hypothesis is based on the progressive loss of cholinergic innervations during AD and was the starting point for using cholinesterase inhibitors to reduce AD symptoms [15–17]. The inflammatory-infectious hypothesis of AD suggests that dysbiosis of human intestinal microflora is responsible for AD [18]. Further, recent findings point to tau as the main factor leading to the development and progression of AD, even proposing that p-tau can accelerate Aβ cleavage from APP [19, 20]. Despite all these hypotheses, AD’s etiology is still largely unknown, and multiple pathways and targets are still under examination.

AD cases have commonly been categorized into early-onset or late-onset AD cases (EOAD and LOAD). This categorization is based on an arbitrary age cut-off set, corresponding to the age at which clinical symptoms start. It is still applied in current research and clinical trials, as recently reviewed by Reitz and colleagues, to determine potential overlap among these categories [21]. EOAD cases represent between 2 and 10% of all AD cases [22]. Patients develop symptoms before the age of 65 and are mostly related to autosomal dominant genetic mutations in APP, situated on chromosome 21 [23], presenilin1 (PSEN1), on chromosome 14 [24, 25] and Presenilin 2 (PSEN2), on chromosome 1 [26, 27]. Heritability of EOAD ranges between 92–100% [28, 29], so some refer to it as familial EOAD. However, some cases follow a non-mendelian pattern of heritability, perhaps due to new rare variants [30]. LOAD has an estimated heritability rate of 60 to 80% [31]. Most LOAD cases are sporadic and present an undetermined familial pattern of disease. LOAD pathogenesis is characterized by a prolonged asymptomatic preclinical phase in which Aβ alterations begin approximately 10 to 20 years before the onset of the symptoms [32].

The most influential genetic risk factor associated with LOAD is the expression of polymorphisms in the apolipoprotein E gene (*APOE*). *APOE’s* role in increasing AD risk is complex and multifactorial. The latest findings revealed that expressing the *APOE2* allele robustly decreases LOAD risk through Aβ-dependent and independent mechanisms [33], whereas the expression of one 4 allele increases threefold the risk of developing AD and expressing two 4 alleles increases risk 9 to 15-fold [34, 35]. The detrimental effects of *APOE* on AD pathology have been linked to its immunomodulatory functions [36]. *APOE* can alter the expression profile of astrocytes and microglia, the key players in the central nervous system’s immune response [37, 38]. *APOE4* exacerbates Aβ aggregation, tau pathology, neuroinflammation, and neurodegeneration [39], but the mechanisms through which *APOE4* exerts its detrimental effects in AD pathology are still under study. Advances in single-cell analysis, transcriptomics, and metabolomic studies have shed some light on the effect of *APOE* upon multiple aspects of AD pathology. In line with this, genome-wide association studies (GWAS) have identified over 44 risk *loci* that influence the risk of suffering LOAD. However, there are still plenty of genetic risk factors remaining uncharacterized. These genetic variants are related to multiple pathways, such as endocytosis and intracellular trafficking, APP processing, tau metabolism, lipid and energetic metabolism, synaptic plasticity, inflammation, innate neuronal immunity, glial cell activation, and proteolytic and transcription processes [21, 40–42].

Thus, this review aims to extensively describe the influence of *APOE* and its polymorphisms in different factors influencing AD, bringing special attention to the latest advances in the AD field regarding *APOE’s* influence in glial activity, synaptic function, cellular processes such as autophagy, and signaling cascades. We also aim to underline the latest information relative to clinical trials in AD, external factors that may influence AD progression, such as diet, sex, and physical exercise. However, the implications of *APOE* are not limited to AD. The *APOE* genotype is also related to the severity of other proteinopathies and neurodegenerative diseases characterized by overt neuroinflammation [43]. Hence, we also aim to comment on the influence of the *APOE* genotype in other neurodegenerative diseases.

## Evolutionary aspects of APOE

Apolipoproteins are well-preserved proteins throughout evolution present in aquatic and terrestrial vertebrates. Apolipoproteins belong to the phylogenetically older and larger protein family that include the Pfam family domain PF01442. This domain is also present in some choanoflagellates, thereby dating the emergence of apolipoproteins earlier than the appearance of animals. From this family, a large number of apolipoproteins emerged. *APOE* orthologs are observed in fish, amphibians, reptiles, and mammals. An analysis of the *APOE* phylogeny comparing 18 species showed that *APOE* differed the most in fish and frogs, indicating significant gene changes occurred in the gene since the divergence from fish and amphibians [44]. Interestingly, *APOE* is absent in birds [45], which additionally lack many of the major proteins involved in cholesterol internalization [46]. This phenomenon is considered an avian-specific loss; since *APOE* is present in alligators (Gene ID: 109,284,755). The apolipoprotein family has undergone numerous gain and loss of function events in the vertebrate lineage [47]. It has been proposed that the *APOE* gene evolved from APOC-1 genome duplication, an event dated approximately 400 million years ago in a common ancestor of fish and mammals [48]. Interestingly, the low-density lipoprotein receptor (LDLR) family, to which apoE binds, is much older and appears to have evolved rapidly after the appearance of multicellular organisms [49]. Humans are the only known species in the animal kingdom with multiple functionally polymorphic variants of *APOE*. Chimpanzees (Pan Troglodytes Verus), our closest evolutionary relatives (divergence approx. 5–7.5 million years ago), appear to have a monomorphic *APOE* gene which is similar to our human *APOE4* [50]. The same happens for Denisovans and Neanderthals, although only a few individual fossils have been sequenced. The *APOE2*, *APOE3*, and *APOE4* variants result from three of the four haplotypes predicted from combinations of the alleles of the two single-nucleotide polymorphisms rs429358, and rs7412. A fourth and extremely rare haplotype consists of the combination of an arginine at position 112 and a cysteine at position 158, and is defined as *APOE1*, also known as *APOE3r*. *APOE1* was described in 3 Italian families [51]. It is still unknown what drove the evolution of the allelic variants. Speculations include that *APOE3* might result from an adaptation to meat-eating since lipoproteins play essential roles in dietary functions [52]. Comparing *APOE* from different species also revealed that species with similar diets cluster more closely, indicating that changes in *APOE* could reflect differing diets of animals [44]. *APOE3* and *APOE2* are also thought to play significant roles in the evolution of human longevity. Humans have exceptionally long lifespans compared with both our primate brethren and mammalian cousins. This increased lifespan presumably evolved in ancestors carrying *APOE4/4*, which is associated with increased mortality and morbidity. Some propose that over millennia the longevity and health of “post-reproductive” individuals increase the fitness of their offspring’s offspring, also known as the “grandmother” hypothesis, which could explain why *APOE3* is now the dominant allele [53]. Some even argue that *APOE3* and *APOE2* were mutations that negate an earlier deleterious mutation fixed in the human line [54]. This hypothesis suggests that chimpanzee *APOE* sequences are most similar to the *APOE4* human allele. However, functionally chimpanzee apoE protein resembles that deriving from the expression of the *APOE3* allele [50]. A possible explanation for this is that humans faced an evolutionary bottleneck at some point in time, and a detrimental mutation occurred in the *APOE4* gene, possibly at position 61, as most mammals, including the great apes, present a threonine molecule at this site [55, 56]. Consequently, the *APOE3* and *APOE2* genes, based on specific amino acid substitutions, are thought to compensate for the detrimental change in human *APOE4*. An alternative hypothesis is that *APOE4* played a more critical role in immune function in an era when infections were the primary selective pressure on humans. Thus, potentially making *APOE4* more prevalent [57]. Supporting this, a study conducted on the forager-horticulturalists Tsimane people suggested that *APOE4* could better preserve cognitive function in the case of high parasite burden compared to *APOE3* [58]. Either way, both simulations and sequence studies strongly suggest that the prevalence of *APOE2* and *APOE3* result of natural selection [59, 60].

## ApoE structure and isoforms

ApoE is a 34 kDa glycoprotein composed of 299 amino acids and belongs to the family of amphiphilic exchangeable lipoproteins. The human *APOE* gene is located in chromosome 19q13.32, constituted by four exons and three introns [61]. The historical progress in understanding apoE structure was recently documented in the review by Chen and colleagues [62]. From the human allelic variations, *APOE* derives in six major genotypes, three of them homozygous (*APOE2/2*, *APOE3/3,* and *APOE4/4*) and three heterozygous (*APOE2/3*, *APOE2/4,* and *APOE3/4*). The resultant proteins differ from each other in the amino acids located at positions 112 and 158, resulting in apoE2 (Cys112; Cys158), apoE3 (Cys112; Arg158), and apoE4 (Arg112; Arg158). The human apoE protein contains three main domains, an N-terminal and a C-terminal domain joined by a flexible hinge region. The N- and C-terminal domains contain the receptor-binding and the lipid-binding regions, respectively [55, 63]. The alterations in the amino acid sequences between apoE isoforms are responsible for the differential protein folding of apoE, leading to the differences in the lipid and receptor binding abilities of the different apoE isoforms [64]. Conversely, the mouse *apoE* gene (*m-apoE*) is located on chromosome 7 and exists as one isoform. Homology between the promoter regions of human and *m-apoE* is less than 40%, and *m-apoE* interactions resemble those of human apoE3 [56, 65]. It is also relevant to note that human and *m-apoE* differ in functionality in most AD processes, such as Aβ clearance, neuroinflammation, and synaptic integrity [66–68]. These disparities justified using the human *APOE* gene sequence in transgenic pre-clinical AD models.

The differential functional properties that confer apoE its polymorphisms urged determining the population’s allelic frequency of *APOE* alleles. The global human allele frequencies of *APOE2*, *E3*, and *E4* are 7%, 79%, and 15% each and result from non-synonymous SNPs located at exon number 4 of the *APOE* gene [69, 70]. More details related to the global distribution of the *APOE* allele frequency in humans can be found in various reviews [71–73]. SNPs in the surroundings of the *APOE* gene can also lead to variations in the apoE CSF levels, such as the *TOMM40* gene, the *APOE* promoter, and distal *APOE* enhancer elements (MEU and BCR) [70]. The case report from Arboleda-Velasquez and colleagues informed about resistance to autosomal dominant AD in a Colombian woman carrying two copies of the Christchurch mutation [74]. The *APOE* Christchurch mutation presents a Ser-136 variant [R136S] [75]. This mutation strongly decreases apoE-LDLR and apoE-heparin-binding [74, 76].

**Fig. 1:**
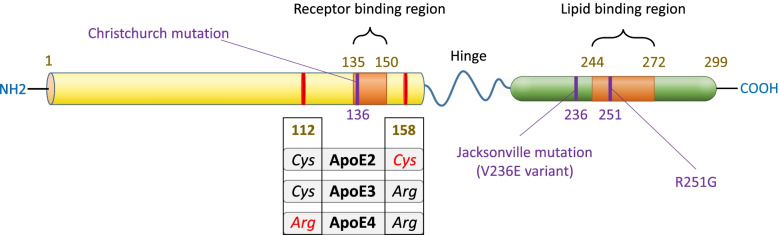
ApoE protein structure and mutations. The human apoE protein is characterized by three main domains, an N-terminal containing the receptor-binding region and a C-terminal domain where the lipid-binding region is located. A flexible hinge region joins N-terminal and C-terminal domains. Mutations at positions 112 and 158 will give rise to the most prevalent apoE isoforms, apoE2, apoE3, and apoE4. Other apoE variants characterized are the Christchurch mutation, which presents a Ser-136 variant [R136S]. ApoE Jacksonville mutation, also called V236E, variant is located at position 236 proximal to the lipid-binding region and reduces apoE self-aggregation. Lastly, the apoE R251G variant induces a single amino acid switch at position 251 and has been initially linked with decreased risk of suffering AD

However, it is interesting to note that heterozygous carriers of the Christchurch mutations seem not to benefit from its protective effects, according to the findings from Hernandez I. and colleagues [77]. Lately, it has been reported the *APOE3*-Jacksonville mutation, which presents an alteration in the lipid-binding region and has been reported to reduce Aβ aggregation in 5xFAD mice [78]. Thus, further studies regarding the influence of this mutation in the development of AD would result in great value (Fig. 1).

## ApoE production, secretion and lipidation in the CNS

### ApoE production and secretion

ApoE production and secretion are highly cell- and tissue-specific, and inducible by various transcription factors and regulatory elements, hormones, cytokines, or lipids [79, 80]. Astrocytes are the major producers of apoE in the CNS, including specialized astrocytes, Bergmann glia, tanycytes, pituicytes, and retinal Müller cells [81]. In addition, microglia, oligodendrocytes, pericytes, the choroid plexus, and neurons under stress situations can also produce apoE to a lesser extent [82–85]. ApoE is produced following the classical secretory pathway; it is synthesized in the endoplasmic reticulum, post-translationally glycosylated and sialylated in the Golgi network, transported to the plasma membrane, and secreted [79, 86]. Multiple studies have focused on the impact of protein glycosylation in AD pathogenesis [87–89], apoE produced by astrocytes is more heavily sialylated and glycosylated, and some studies suggest the existence of tissue-specific apoE glycoforms [79, 90].

ApoE expression and secretion by astrocytes are regulated by the liver X receptor (LXR) and the retinoid X receptor (RXR), DNA-binding factors implicated in cholesterol metabolism. LXR has also been related to the immune response during inflammation and blood–brain barrier (BBB) function [91–93]. Brain-derived neurotrophic factor (BDNF) can stimulate LXR to induce *APOE* expression and astrocytic cholesterol efflux [94]. Recently, Axl receptor tyrosine kinase was also found to regulate *APOE* in human astrocytes [95]. LXR and RXR also are involved in the transcriptional regulation of *APOE* [96]. RXR is primarily expressed in hepatocytes, even so, it has been related to brain clean-up after stroke, and RXR agonists have been proposed as a therapeutic approach for AD treatment as are directly targeting LXR and RXR [97–99].

**Fig. 2:**
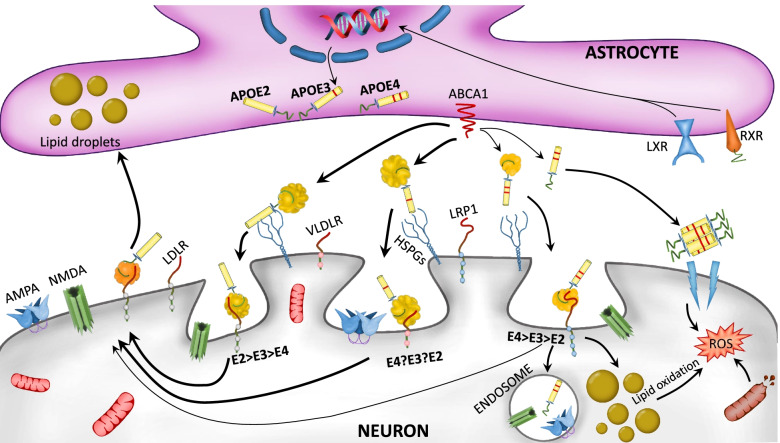
Roles of distinct *APOE* isoforms in astrocyte-neuron communication. The liver X receptor (LXR) and retinoid X receptor (RXR) activation regulate *APOE* isoforms expression in astrocytes. ATP-binding cassette transporter (ABCA1) is responsible for apoE lipidation and secretion to the extracellular space, so as to lipidate apoE already present in the extracellular space. ApoE can be later on recognized by several neuronal lipid receptors including VLDL-receptor (VLDLR), LDLR, Low-density lipoprotein receptor-related protein 1 (LRP1), and heparan sulfate proteoglycans (HSPGs). The distinct apoE isoform lipidation status promotes distinct receptor affinities. The apoE4 isoform presents alterations in its physiological pathways compared to other isoforms, such as low lipidation status, the formation of non-lipidated apoE4 aggregates, and a poor lipid turnover towards astrocytes, which leads to neuronal lipid-droplet accumulation. This promotes mitochondrial damage and reactive oxygen species (ROS) production. Moreover, apoE4 promotes endosome formation and degradation of synaptic receptors like AMPA or NMDA, leading to impaired synaptic function

Additionally, ATP binding cassette transporter A1 (ABCA1) is transcriptionally regulated by LXR [100]. ABCA1 is one of the main transporters involved in cholesterol efflux regulation through lipoproteins. ABCA1 plays a crucial role in forming high-density lipoproteins (HDL) [101, 102]. It has been demonstrated that the higher tendency of apoE4 to self-aggregate decreases ABCA1 membrane-recycling and therefore leads to lower lipidation of apoE4 particles (Fig. 2) [103].

The brain is the richest body organ in cholesterol, bearing approximately 20% of total body cholesterol, and apoE is the primary responsible for lipid transport and maintenance of cholesterol homeostasis in the brain. ApoE is essential in providing neurons with cholesterol and carrying out excessive cholesterol clearance [104, 105]. ApoE is involved in cell membrane support, synaptic plasticity, signal transduction, proteostasis immunomodulation, and repair after an injury [106–108].

### ApoE lipidation

To perform its correct functions, apoE must be secreted and lipidated. ApoE lipidation is mediated by cell-surface receptors, ABCA1 and ATP Binding Cassette Subfamily G Member 1 (ABCG1), which moderate cholesterol efflux to apoE [109]. ABCA1 and ABCG1 are expressed in neurons, glial cells, and macrophages [110, 111]. It has been demonstrated that *APOE4* promotes greater expression levels of ADP-ribosylation factor 6 (ARF6), a protein involved in ABCA1 recycling, by sequestering ABCA1 in the late endosomes [103]. The receptor-binding abilities, molecular stability, and correct functioning of apoE are conditioned by its lipidation status. Isoform variations of *APOE* will directly impact its lipidation (apoE4 < apoE3 < apoE2) [112, 113]. In vitro model results pointed out that apoE lipidation prevents its self-aggregation [112]. Thus, due to the critical impact of lipidation on apoE’s physiological and pathological roles, increasing apoE lipidation has been proposed as a target for AD treatment [114].

Lipidated apoE will be internalized through apoE receptors, e.g., low-density lipoprotein receptor-related protein 1 (LRP1) and LDLR [71, 115]. LDLR belongs to the LRP family, seven structurally close-related transmembrane proteins that include LRP1, LRP1b, LRP2, LRP4, LRP8, VLDLR, and LDLR [116]. Previous findings reported that LRP1 preferentially binds to recombinant apoE, lipoprotein particles enriched for apoE, and HDL particles derived from CSF; Whereas LRPs and VLDLR bind all apoE particles, including lipid-free apoE in the case of VLDLR, LDLRs preferentially bind to lipidated apoE particles [117–119]. Apolipoprotein E receptor 2 (ApoER2), also known as LRP8, and VLDLR are core components of the Reelin signaling pathway implicated in regulating learning, neuronal migration, dendritic growth, and synaptic plasticity [120–122]. VLDLR mRNA expression levels are the highest in the cortex and the cerebellum, particularly in astrocytes, microglia, oligodendrocytes, pyramidal neurons, neuroblasts, glioblasts, and Cajal-Retzius cells, whereas ApoER2 expression in the CNS is related to the brain areas such as the cerebellum, hippocampus, olfactory bulb, neocortex, and cortical neurons, especially in granule cells of the dentate gyrus and the pyramidal cells [123, 124].

## Impact of apoE isoforms on receptor and target binding

Allelic variation of *APOE* will influence the lipid particle size that apoE binds to, the type of lipids and proteoglycans that apoE will interact with, the brain lipid profile, and apoE’s interacting abilities with its receptors. For instance, a study comparing apoE particle size from temporal lobe brain homogenates of AD and control patients by size-exclusion chromatography found that apoE particles in *APOE4/4 patients* were twice as big as those from *APOE3/3 AD* patients and *APOE3/3* control subjects [125]. However, this study did not include genotype control *APOE4/4* subjects, and no comparison was performed between *APOE4/4* and *APOE3/3* control subjects. The increased particle size in *APOE4/4* subjects might be associated with the fact that the *APOE4* genotype is related to high blood LDL-cholesterol levels [126].

It has been proposed that only monomeric apoE can bind lipid vesicles and that phospholipids play a role in successful apoE-receptor interaction [127, 128]. ApoE4 peptides present a high lipid-binding affinity related to a lower recycling capacity and increased intracellular cholesterol accumulation [129, 130]. Combining chemical cross-linking with mass spectrometry, Henry N. and colleagues showed that apoE4 adjusts its conformation for its recognition by LDLR [131]. ApoE4 can also bind LRP1 with greater affinity than apoE3 and apoE2 [132]. ApoE2 is characterized by reduced binding affinity to LDLRs, which relates to the high susceptibility of *APOE2/2* carriers to develop type III hyperlipoproteinemia [133]. Nonetheless, recent meta-analysis studies have also revealed *APOE4* as a hyperlipidemia risk factor [134].

Targeted lipidomic analysis in *APOE* knock-in mice have evidenced a higher susceptibility of lipidic alterations in the entorhinal cortex (EC) of *APOE4* mice [135]. Shotgun lipidomic studies in the parietal lobe of AD brains from *APOE2*, *APOE3* and *APOE4* carriers evidenced significantly lower phospholipid levels in *APOE4* carriers and marked differences in the brain lipidomes between *APOE3*/3 and *APOE4* carriers [136]. Posterior studies have showed an upregulation of several phospholipid classes in *APOE3/3* human brain samples compared to *APOE4/4* carriers [137].

Regarding apoE’s lipid-binding abilities, apoE4 presents a binding preference for VLDLs and LDLs, while apoE3 presents a higher affinity towards HDLs [138]. ApoE3 and apoE2 bind HDL with higher affinity than apoE4, whereas apoE4 strongly binds VLDLs [139, 140]. Frieden and colleagues proposed a mechanistic explanation for the functional differences in lipid binding between apoE3 and apoE4 based on their full-length monomeric structure and their domain-domain interactions using molecular dynamics [141].

ApoE can also bind proteoglycans and glycosaminoglycans [142]. ApoE4 binds more strongly to heparan and dermatan-sulfate than apoE2 and apoE3 [143]. However, lipid-free apoE4 tends to bind to heparin to a lower extent than lipid-free apoE3 and apoE2, whereas lipidated apoE isoforms tend to bind to heparin in a similar manner [62, 144]. Both lipidated and non-lipidated apoE bind heparan sulfate proteoglycans (HSPGs) through the N-terminal binding site to clear remnant lipoproteins, particularly in the liver [117, 145–147]. The extracellular matrix is very rich in HSPGs, which have been proposed as a therapeutic target in tauopathies due to their tendency to associate with tau aggregates [148]. HSPGs are also related to neuroinflammation and colocalize with the core of amyloid deposits [149, 150]. HSPGs can bind Aβ and facilitate its oligomerization and aggregation [151]. In vitro studies have proposed that the interaction between HSPGs and LRP1 mediates Aβ uptake by primary neurons [152]. Moreover, genetically engineered animal models lacking HSPGs by conditional deletion of the *Ext1* gene show lower Aβ deposition and higher clearance [153]. A summary of the identified apoE receptors is reflected in Table 1. The interacting affinities of the apoE isoforms with lipid receptors and its molecular targets are depicted in Tables 2 and 3.

**Table 1:** Interacting receptors with apoE and CNS cell types in which they can be expressed

Receptors interacting with apoE	CNS cell-type
SORLA (SORL1, LR11)	Microglia [154], Neurons [155, 156]
VLDLR	Astrocytes, microglia, oligodendrocytes, pyramidal neurons, neuroblasts, glioblasts, Cajal-Retzius cells [123]
ApoER2 (LRP8)	Granule cells of dentate gyrus, piramidal cells [123]
LDLR	Astrocytes, neurons, oligodendrocytes [157, 158]
LRP1	Neurons, astrocytes, microglia, [159, 160]
LRP2 (Megalin)	Oligodendrocytes [161], astrocytes [162], neurons [163]
LRP4 (MEGF7)	Astrocytes, oligodendrocytes, microglia [164], neurons [165]
LRP5	Microglia [166], astrocytes [167], neurons [168]
LRP6	Microglia [166], astrocytes [169], neurons [170]
TREM2	Microglia [171], dendritic cells [172], astrocytes [173]
TLR4	Microglia [174], astrocytes [173], neurons [175]

Results retrieved from human, animal and in vitro studies. () Other names by which the receptor is also known

**Table 2:** Described binding affinities of the apoE isoforms to the lipoprotein-binding receptors LDLR, LRP1, VLDLR, and the endocytic receptor SORLA

Receptor	Binding affinity			Highest binding affinity to
	apoE2	apoE3	apoE4	
**LDLR**	↓↓	↑↑	↑↑	Lipidated apoE particles [119]
**LRP1**	↓	↑	↑	Recombinant apoE and apoE aggregates [119]
**VLDLR**	-	-	-	Lipid-free apoE [118]
**SORLA/LR11**	↓	-	↑	Not well defined [156]

SORLA presents common structural features with the LDL receptor family. (-) no differences observed between apoE isoforms, ↑ (high), ↑↑ (higher), ↓ (low), ↓↓ (very low)

**Table 3:** ApoE isoform lipid interacting abilities through the lipid binding domain and the N-binding domain

ApoE isoform	Lipid binding domain	N-binding domain
apoE4	VLDLs, LDLs [138]	Heparan and Dermatan sulfate [143] Heparin (↓ lipid free apoE4, lipidated apoE4 [144]
apoE3	HDL [176] LDL [118]	Heparin (lipid free apoE3) [144]
apoE2	HDL [176] ↓ LDL [177]	Heparin (lipid free apoE2) [144]

↓ (low binding affinity)

## Impact of *APOE* polymorphisms on CNS cell physiology

ApoE isoforms can affect multiple cellular functions in various cell types of the CNS, such as synaptic function in neurons, lipid homeostasis in astrocytes, and immune responses in microglial cells [178]. ApoE isoforms also affect mitochondrial biogenesis and dynamics [179]. The study by Area-Gomez and colleagues observed alterations in the mitochondrial respiration process among the distinct *APOE* genotypes, such as upregulation of genes related to oxidative phosphorylation decreases in mitochondrial respiration in the cortex and hippocampus of aged female and male *APOE4/4* mice *versus APOE3/3* [180]. Inorganic polyphosphate (Poly-P), proposed as an alternative to ATP for cellular energy storage, is involved in mitochondrial function and related to the mitochondrial processes affected in AD, such as calcium homeostasis alteration and acceleration of amyloid fibril formation [181]. ApoE was identified as a Poly-P ligand; however, the differential effect of apoE isoforms and their interaction with Poly-P are yet to be elucidated [182, 183]. *APOE4* is closely related to synaptic degeneration, as observed from studies with cerebral organoids from AD patients carrying *APOE4,* in which *APOE4* carriers presented higher levels of cellular apoptosis and decreased synaptic integrity [184]. Studies performing RNA-seq in iPSC neurons and glial cells have reported dramatic switches in the transcriptomic profile when performing isogenic conversion of *APOE4* into *APOE3* and a reduction in the *APOE4*-related phenotype [178, 185].

## Distribution of apoE in the brain, cerebrospinal fluid, and blood

Over the past years, the potential diagnostic value of knowing apoE levels in biological fluids has gained substantial interest. So as to evaluate the impact of *APOE’s* allelic isoforms on the fluid concentrations of apoE in the cerebrospinal fluid (CSF) and plasma. As one of the main lipoproteins in the CSF, apoE preferentially binds HDLs and transports cholesterol within the CNS [104, 186]. The impact of the *APOE* genotype on the transport of HDL particles, cognition and its connection with the development of neurodegenerative diseases has been reviewed by other authors [187, 188].

Studies examining apoE protein levels in the brains of *APOE* knock-in mouse models have observed a genotype-dependent decrease in apoE levels apoE2 > apoE3 > apoE4 [189–191]. In the case of targeted replacement *APOE3/4* mice, apoE4 constitutes 30 to 40% of total apoE levels, and newly synthesized apoE4 presents an enhanced degradation and reduced half-life compared to apoE3 [189].

Regarding the expression pattern of apoE in different brain areas, Donald Schmechel’s group analyzed apoE protein distribution in the cerebellum, frontal cortex, and hippocampus of *APOE* targeted-replacement mice, which revealed significant regional differences but no difference between the *APOE* genotypes in the apoE regional distribution [192]. The cerebellum was the area with the highest apoE expression, and the hippocampus the lowest. The authors also compared plasma apoE levels between *APOE2*, *APOE3* and *APOE4* transgenic mice and found that plasma apoE2 levels were 16-fold higher than the other two isoforms [192]. However, these findings contrast with those in Non-Obese Diabetic (NOD) background (FRGN) humanized liver mice in which they observed that the highest brain apoE levels (endogenous mouse apoE) were found in the hippocampus and the lowest in the thalamus [193]. Furthermore, these authors also found that the brain tissue levels, specifically in the cortex and to some extent in the hippocampus, are lower in mice with humanized *APOE4* livers versus those with non-*APOE4* livers [193]. Additional studies from *APOE* targeted replacement mice have shown that mice expressing apoE4 present lower CSF apoE levels than the other isoforms [189, 190, 194].

Plasmatic and CNS apoE do not cross the BBB, thus constituting two independent apoE pools; Peripheral apoE will be primarily produced by hepatocytes, whereas in the CNS, astrocytes will be the major apoE producers [195, 196]. *APOE* knock-in mice studies evaluating the influence of hepatic apoE on Aβ deposition observed that genetic deletion of hepatic *APOE* did not influence brain apoE levels but induced plasma lipid profile changes and a decrease in plasmatic apoE levels [197]. Lane-Donovan and colleagues evidenced that genetic restoration of plasmatic apoE into wild-type levels in *APOE* knock-out mice normalized plasma lipids [107]. Furthermore, while restoring plasmatic apoE levels in the prior study did not rescue synaptic loss, it completely restored learning and memory in mice [107]. Thus, suggesting that both CNS and plasmatic apoE are independent parameters impacting brain health. Interestingly, results derived from humanized-*APOE4*-liver mice recently evidenced a negative impact of hepatic *APOE4* in synaptic integrity, insulin signaling and increased neuroinflammation coupled to lower brain apoE4 expression [193]. Therefore, the potential negative contribution of peripheral apoE4 to the development of pathological brain processes should not be ruled out.

Studies of apoE levels in human CSF and plasma have shown results not always in line with those found in mice. For instance, some human studies found that the *APOE* genotype does not influence the CSF apoE levels [198, 199]. Nonetheless, the latest findings further point out a difference in the CSF apoE isoform composition in *APOE* heterozygous subjects [199–201]. Nonetheless, neither the total CSF apoE concentration nor its isoforms were related to Aβ status nor the degree of dementia of the AD patients, in agreement with prior studies [199, 200, 202].

A recent publication from Berger and colleagues evaluated the proteomic changes in the CSF of AD Neuroimaging Initiative (ADNI) samples by applying linear regression models adjusted by age, sex, *APOE4* copy number, and linear models to adjust by AD clinical status or CSF levels of Aβ or tau. The authors observed that increasing *APOE4* copy number was related to decreased CSF levels of C-reactive protein (a classical inflammatory marker), which correlated with cognitive impairment and AD progression [203, 204]. The study by Cruchaga and colleagues examined the correlation between CSF and plasma apoE levels in 641 AD patients and controls, in which they found a very low correlation [205]. However, it was observed that men presented significantly lower plasmatic apoE levels than women [205]. The prior findings correlate with Nielsen and colleagues who demonstrated higher apoE4 levels in females versus males [206]. Nielsen and colleagues also observed an age-dependent increase in the apoE3 isoform levels only in females [206]. Posterior studies from the same group found that, specifically in males, plasma apoE3 levels were negatively correlated to plasma glucose levels which were associated with brain glucose metabolism [207]. Hence, these findings could be related to the higher incidence of AD in women and the link between low plasma apoE levels and an increased risk of suffering dementia [208].

Human studies have identified low plasma apoE values as a significant risk factor for AD and other types of dementia [209]. Low plasma apoE levels described in *APOE4* carriers resulted from a specific lowering of the apoE4 isoform [199, 206]. Whether plasma apoE levels are relevant to brain processing mechanisms remains controversial [195]. Nevertheless, a higher ratio of plasma apoE4 to apoE3 isoform levels in cognitively healthy *APOE3/4* carriers was associated with reduced glucose metabolism in the hippocampus and gray matter volume reductions in several brain areas [206]. Low plasma apoE levels negatively correlate with cognition and CSF biomarkers [210]. Notably, whereas CSF apoE levels did not appear to be affected by the *APOE* genotype or AD diagnosis, some studies suggest that amyloidosis is associated with reduced CSF apoE levels in an *APOE4* genotype-dependent manner [199–201]. Interestingly, the fluid composition of apoE isoforms in *APOE* heterozygous individuals differs between plasma and CSF, where for example, *APOE3/4* subjects exhibit a different apoE4/apoE3 ratio in both compartments [199, 201, 206]. Lastly, it must also be noted that there are significant differences between plasma and CNS apoE turnover rates, supporting the idea that the pathways implicated in apoE metabolism differ between the CNS and the periphery [211].

## APOE in the neuroinflammatory response

### Neuroinflammation

Inflammation in the CNS, also called neuroinflammation, is a short-term body reaction to a noxious stimulus. Neuroinflammation aims to repair brain damage and restore brain homeostasis. Thus, it has short-term beneficial effects, yet excessive and unrestrained neuroinflammation is deleterious [212, 213]. Multiple factors can trigger neuroinflammation, such as traumatic brain injury, stroke, high-fat diet, exposure to drugs of abuse or aging [214–217]. Chronic neuroinflammation is a common characteristic of numerous neurodegenerative diseases, including Alzheimer’s disease, in which chronic neuroinflammation has become the third hallmark of its pathogenesis [218]. During neuroinflammation, the abnormal production of pro-inflammatory cytokines will trigger multiple signaling pathways [219, 220]. The two key cell types driving neuroinflammation in the CNS are microglial cells and astrocytes [221].

Microglial cells constitute the brain-resident macrophages of the CNS. They represent 10% of the cell population in the CNS and are the first-line defense against pathogenic agents or brain damage. Microglial activation will vary according to the type of stimulus, the intensity, and context, and under physiological conditions, they perform constant surveillance of the microenvironment in a very dynamic way through a vast array of proteins and receptors defined as the sensome [222, 223]. Some of the physiological roles in which microglia are involved are neurogenesis, synaptic pruning, or synaptic plasticity [224–226]. There is a close relationship between microglial morphology and function [227, 228]. Traditionally, microglia were categorized into two different phenotypes, M1 phenotype (classical activation) and M2 phenotype (alternative activation). Nowadays, this dichotomic classification is obsolete, and there is evidence that microglia can display mixed profiles in vivo and may also be regionally heterogeneous [229, 230]. In response to a noxious stimulus, microglia will suffer a series of morphological changes and induce an increase in the expression of surface receptors and the release of pro-inflammatory cytokines. Prolongation of this inflammatory process will compromise microglial cells’ homeostatic functions, including the maintenance of the BBB integrity [231, 232].

When comparing across species, Sullivan and colleagues observed a glial pattern of *APOE* expression in humans, African green monkeys, and knock-in *APOE* mice [192]. In humans, *APOE* isoforms will impact glial activation and migration and synaptic function [233, 234]. *APOE4* knock-in mice present increased glial activation after LPS insult, increased production of pro-inflammatory cytokines, and an extensive loss of synaptic proteins compared to *APOE3* and *APOE2* knock-in mice [235, 236].

Even though astrocytes are the principal producers of apoE in the brain, microglial cells can also act as an apoE source under specific circumstances; for example, a recent publication from William Rebeck’s group evidenced that astrocytes secrete two differentially glycosylated apoE species, whereas microglia secrete a single apoE species while containing two intracellular apoE forms, the three of them glycosylated [237]. Primary cultures of glial cells from *APOE* knock-in mice studying the microglial and astrocytic response after priming with exogenous TNF-α or LPS showed that TNF-α reduces cellular expression and secretion of apoE in *APOE4* astrocytes [237]. In contrast, LPS insult did not produce changes in astrocytic apoE levels. Nonetheless, *APOE3* and *APOE2* microglia showed increased secretion of apoE after LPS treatment, whereas no effect was observed in *APOE4* microglia. Thus, suggesting that *APOE4* astrocytes and microglia present dysfunctional responses towards inflammatory insults [237].

However, neuroinflammation is a complex process that will eventually entwine all the cells in the CNS. During neuroinflammation, activated microglia release pro-inflammatory cytokines like tumor necrosis factor-α (TNF-α), interleukin 1-β (IL1-β), IL-6, IL-12, interferon-γ (IFN-γ), and molecule C1 from the complement system (C1q) to their environment [218]. Additionally, innate and adaptative immune responses will also affect neuroinflammation. Immune cells such as T cells, CD4^+^ cells and B cells can cross the BBB and contribute to the neuroinflammatory process [238]. ApoE can influence immune responses by multiple mechanisms; for instance, in vitro and mouse studies have evidenced the lymphocyte modulatory action of apoE [239, 240]. ApoE also promotes neutrophil activation [241] and mediates antigen presentation on natural-killer cells [242]. Macrophages will also infiltrate through the BBB during neuroinflammation [243, 244], and it has been demonstrated that LPS can repress *APOE* gene expression in macrophages through the Tpl-2/MEK/ERK pathway [245]. Interestingly, even if the implications of adaptative immunity in AD are still largely unknown, promising small-scale studies suggest the implication of specialized CD8 T cells linked to the AD neurodegenerative process [246]. Therefore, it is vital to determine the molecular mechanisms comprising apoE and immunity in the neuroinflammatory process.

### Microglial receptors and their interaction with apoE

As mentioned in the previous section, microglia constantly analyze their surrounding environment through the sensome [223]. Since macrophages and microglial cells share a common origin, they both express macrophage markers, such as F4/80, CD11b, CX3Cr1, Iba1, and CD45 [247–249]. Additionally, as the main antigen-presenting cells of the CNS, microglia express in their surface MHC proteins, which allow antigen presentation to CD8 + and CD4 + T cells (cytotoxic and helper T cells) [250]. Whereas MHC I proteins are ubiquitously expressed, microglial expression of MHC II proteins is considered a marker of neurodegeneration and has been related to AD [251, 252]. A study investigating the effects of the human MHC II (HLA) and its relationship with *APOE* genotype, revealed that the mutation DRB1*13 in human HLA-II was found to be protective against age-related changes in the neural network functioning and presented a similar effect as to carry *APOE2* allele [253].

Among the collection of receptors present in microglia, receptors able to recognize stimuli of quite different nature are known as pattern recognition receptors (PRRs), which are located, with some exceptions, on the plasma membrane of microglia. The main PRRs include toll-like receptors (TLRs), inflammasome-forming nucleotide-binding oligomerization domain (nod)-like receptors (NLRs), the receptor for advanced glycation end-products (RAGE), and triggering receptor expressed on myeloid cells 2 (TREM2) [254, 255]. Concerning PPR’s ligands, the microglia field has consistently used the terminology pathogen-associated molecular patterns (PAMPs) and danger-associated molecular patterns (DAMPs). DAMPS include released or secreted molecules from injured neurons or other cell types [230]. However, the term neurodegeneration-associated molecular patterns (NAMPs) recently rose to describe the danger signals present during brain disease conditions. Likewise, it must be highlighted that while classical DAMPs have traditionally been associated with binding to different PRRs, among other receptors, NAMPS can also activate TREM2 and further drive the acquisition of the disease-associated microglial phenotype (DAM) [256, 257]. However, regardless of these definitions, DAMPS and NAMPS co-exist in the diseased brain, thus giving rise to complex microglia polarization states. Indeed, recent massive transcriptomic analyses have demonstrated that microglia with different transcriptomic profiles co-exist under various disease conditions [258], thus overcoming the old simplistic view that microglia polarize into two opposite polarization states (pro-inflammatory and anti-inflammatory, or tumor-supportive phenotype) [259].

Without a doubt, TREM2 is more than a rising star in the microglia field, whose attention has exponentially increased since it was identified as a risk gene for AD (for reviews, see [255, 260]). In the brain, TREM2 is essentially expressed by microglia [255], and consequently, elucidation of microglia-associated roles of TREM2 and the implications of the *APOE* genotype under disease conditions is a considerable challenge in the field. Interestingly, upregulated TREM2 mRNA expression levels have been detected in human isolated-microglial cells from AD patients [261, 262]. A remarkable finding was the identification of a rare variant of TREM2 (R47H) as a robust risk gene of AD by two independent studies, followed by the identification of an additional variant (R62H), thus, supporting the significant role of TREM2 in microglia biology associated to neurodegeneration [263, 264]. At that time, Zhang and colleagues evidenced that TREM2 and protein tyrosine kinase binding protein (tyrobp) (also known as DAP12; the adaptor/signaling partner of TREM2) are strongly associated with LOAD pathophysiology. The relevance of the TREM2/DAP12 axis is illustrated by the fact that over 40% of sensome proteins are somewhat related to DAP12 [223]. Typical TREM2 ligands include phosphatidylserine present in apoptotic cells, glycolipids, sphingomyelin, and sulfatide present in damaged myelin. However, other beckoned ligands include apoE and clusterin (CLU/ApoJ), Aβ itself, phosphatidylinositol, and phosphatidylcholine [255, 260]. Besides, it has been demonstrated the ability of galectin-3 (gal-3) to bind to and stimulate TREM2 [265]. TREM2 senses the environment under disease conditions through an extracellular domain that belongs to the immunoglobulin superfamily [266]. TREM2 interacts with two different adaptor proteins upon ligand binding, DAP12 and DAP10. DAP12 activates spleen tyrosine kinase (SYK) and triggers downstream pathways involved in survival, migration, proliferation, activation, and phagocytosis, whereas DAP10 leads to Phosphatidylinositol 3-kinase (PI3K) recruitment and subsequent phosphorylation cascade that promotes Ca^2+^ mobilization, integrin activation, cytoskeleton rearrangement, mechanistic target of rapamycin (mTOR) and mitogen-activated protein kinase (MAPK) signaling [266].

The significance of TREM2 in governing microglia activation states has been validated thanks to the advent of massive transcriptomic analysis at the single-cell level. Keren-Shaul and colleagues identified a microglia subpopulation displaying a unique molecular signature in 5xFAD mice (a transgenic mouse model of AD) [267]. This microglial phenotype was named “disease-associated microglia” (DAM) and is characterized by downregulation of essential homeostatic genes, including *P2ry12, Cx3cr1,* and *Tmem119*, along with upregulation of selective genes including *apoE, Itgax Ctsd, Igf1, Lpl, Clec7a, Tyrobp,* and *Trem2* [267]. The authors identified two clusters of activated microglia in 5xFAD mice that were not present in WT mice. It was suggested that one of the clusters corresponded to an intermediate state (Stage 1 DAM) between homeostatic microglia and DAM (stage 2 DAM) [267]. Mechanistically, the authors concluded that the switch from homeostatic microglia to stage 1 DAM was TREM2-independent, while the shift from transition microglia to DAM was TREM2-dependent [267]. Interestingly, apoE was found to be involved in the TREM2-independent step together with tyrobp [268].

In an independent study, Krasemann and colleagues analyzed single-cell microglial transcripts from animal models of neurodegeneration, including AD, ALS, and MS [269]. This study revealed a strikingly similar transcriptional network across the different disease conditions tested and evidenced, similar to DAM, loss of homeostatic genes (e.g., *P2ry12*, *Tmem119*, *Gpr34*, *Csf1r*, *Hexb,* and *Mertk*), and upregulation of genes such as *apoE, Spp1*, *Itgax*, *Lgals3*, *Axl*, *Clec7a*, and *Ccl2 *[269]. This microglial phenotype was named microglia neurodegenerative phenotype (MGnD) and demonstrated critical roles of both TREM2 and *apoE* in driving MGnD transcriptional profile [269].

It remains somehow paradoxical that DAM microglial cells are suggested to be neuroprotective, whereas MGnD are neurotoxic [267, 269]. The protective/harmful role of microglia is probably associated with the context, a view notably exemplified by TREM2 signaling. Thus, the absence of TREM2 in AD mice leads to increased neuritic dystrophies associated with Aβ plaques [270, 271]. In contrast, studies in tau transgenic mice have rendered conflicting results regarding the role of TREM2 in tau pathology, including a reduction and exacerbation of the pathology [272, 273]. Another set of studies has highlighted the prominent roles of TREM2 in the seeding/spreading of both Aβ and tau pathologies [274, 275]. In a seeding model based on intracerebral injection of Aβ-rich brain extracts from AD patients or aged APP mice, the absence of TREM2 led to increased seeded amyloid plaques exhibiting low levels of microglia-derived apoE deposition in amyloid plaques in mouse brains [274]. In line with these observations, reduced apoE in amyloid plaques was found in the brains of AD patients carrying different TREM loss-of-function variants [274]. These observations could be associated with reduced microglia clustering to Aβ plaques associated with TREM2 deficiency and the subsequent inability to develop a DAM program [267] and again highlight that microglia may dictate quite polarized directions at the crossroads of Aβ and tau pathologies.

Deeply related to the *APOE* context, TREM2 signaling is critical for the efficient expression of genes required for lipid metabolism in human microglia, a process dependent on phospholipase CY2 (PLCϒ2) signaling [276, 277]. PLCϒ2 signaling can also act downstream of TLR activation, and it has been proposed that this important node may enable microglia to elicit either a polarization state through TREM2 activation or a pro-inflammatory response through TLR activation [276]. This research group also observed that a TREM2 loss of function leads to aberrant lipid metabolism and enhanced pro-inflammatory response. In parallel, other authors have also observed that TREM2 deletion decreases microglial cell survival and impairs microglial phagocytosis of apoE [276, 278]. Thus, in keeping with a switch from a beneficial to a neurotoxic pro-inflammatory phenotype, both TREM2 and PLCϒ2 are AD risk genes [279]. Regarding ApoE’s interaction with TLRs, it has been described a deleterious effect of the APOE4 genotype in AD through the TLR4-dependent pathway [269], and apoE3 can inhibit macrophage activation driven by TLR4 [280], TLRs recruit TIR domain-containing adaptor proteins such as MyD88, TRIF, TIRAP/TRAM, or TRAM, leading to activation of different signal transduction pathways, including NF-κB and MAP kinases and hence inducing inflammatory cytokine gene expression [281]. Upon ligand binding, it is well known that TLR activation primes microglia, the first step for effective inflammasome activation [282]. This step includes expressing NLRP3 and pro-IL-1β in an NF-κB dependent manner [282]. TREM2 will negatively regulate TLR-induced cytokine production [283]. The interaction between apoE and inflammasome activation has been described in peripheral lavage fluid macrophages, but no interaction has been yet reported between microglial NLRP3 and CNS apoE [284]. However, Wong and colleagues have observed in human AD brains and transgenic mice tissue that 25-hydrocholesterol, a relevant inflammatory mediator produced by microglia, promotes IL-1β mediated neuroinflammation in an apoE isoform-dependent trend (apoE4 >  > apoE3/apoE2), and apoE4-expressing microglia produce higher levels of 25-hydrocholesterol [285].

Another receptor family that has gained growing relevance are TAM receptors, including tyro3, axl and mer [286, 287]. These receptors have long been associated with the phagocytic clearance of apoptotic cells [288]. Homeostatic microglia express high gene levels of macrophage efferocytosis receptor c-Mer tyrosine kinase (Mertk) [267]. However, Axl is highly upregulated in the DAM phenotype, including plaque-associated microglia [256]. In vitro models have also shown Axl as a potential regulator of apoE homeostasis in astrocytes [95]. In APP/PS1 mice lacking TAM receptors, transcriptomic response to Aβ plaques was blunted, including a downregulation of genes related to lipid metabolism, including *apoE* [289]. Overall, this study suggests that microglial TAM receptors recognize and phagocytose Aβ plaques to further promote the formation of dense-core plaques [289]. Hence a prominent role of apoE in the formation of dense-core plaques is plausible in line with the well-accepted function of *APOE4* in amyloidogenesis, enhancing fibrillization and compaction [290, 291].

In prior sections of this manuscript, we have addressed some of the relevant functions of the LRP1 receptor and its interaction with apoE. LRP1 is an endocytic and signal transduction-related cell surface receptor. Aside from being expressed in microglia, it is also highly expressed in neurons and to a lesser extent in neuroblasts, radial glia, astrocytes, and oligodendrocyte progenitor cells (OPCs) [292]. In-vitro primary microglial cell studies showed that LRP1 activation suppresses microglia activation through modulating JNK and NF-kB signaling pathways [293, 294]. On the contrary, results derived from spinal cord-derived primary microglial cultures showed that LRP1 plays a significant role in microglial cell activation in response to LPS insult and amplifies neuroinflammatory signaling pathways [295]. It has also been proposed that LRP1 mediates apoE’s effect on microglial inflammation [296]. Similarly, it was recently observed in animal models of AD that brain LRP1 silencing increased the inflammatory response by the TLR4/NFκB/MAPKs signaling pathway [297].

It is worth noting that microglial cells constitute the major source of the complement system component C1q. The complement cascade comprises soluble and membrane-bound proteins and represents the section of the immune system devoted to pathogen and cellular debris recognition and synapse elimination during developmental and adult stages [298, 299]. It has been demonstrated that all apoE polymorphisms inhibit the classical complement cascade by acting in the early activation stages by binding with high affinity to C1q. C1q-apoE complexes formation correlates with cognitive decline and suppression of complement protein C5 ameliorated inflammation [300].

### *APOE* and microglial dynamics

The phenotypic analysis of microglia has become a hot topic in recent past years and highlighted the heterogenicity of microglial cells across the brain.

Recent advances in transcriptomic, bulk-RNA-seq, single-cell RNA-seq, and imaging techniques have underlined the significance of studying microglial dynamics during inflammatory pathologies. Albeit some of these techniques have led to conflicting results, they have allowed relating each microglial phenotype with a particular molecular signature to some extent, a previously reviewed by multiple authors [227, 229, 258, 301, 302]. However, establishing a direct link between microglial phenotypes and their specific functions remains challenging, as evidenced by other authors [229, 303].

The highest microglial heterogenicity has been observed at early postnatal time points, particularly in white-matter brain areas, such as the cerebellum and the Corpus Callosum [301]. Interestingly, some immature sub-populations of microglia are characterized by high gene-expression levels of *APOE* [304]. A review covering developmental microglial dynamics was performed by Menassa and colleagues [305].

Under non-pathological conditions, the so-called resting state microglia are characterized by highly ramified processes and a small rounded soma, together with the gene expression of homeostatic microglial markers, such as *P2ry12*, *Tmem119*, and *Sall1* [223, 306–308]. Aside from homeostatic microglia, intermediate activation-state microglia, DAM, and MGnD microglial phenotypes described by Keren-Shaul, Krasemann, and colleagues, additional transcriptional microglial phenotypes have been recently identified [267, 269]. For instance, single-cell sequencing from white and grey matter led to the characterization of White-matter microglia (WAM), which are relevant in the AD context because the brains of AD patients present a high white matter loss, which is also related to motor and cognitive dysfunction [309–311]. Prior studies have shown that *APOE4* can modulate white matter integrity [312]. It has been proposed a link between *APOE4* polymorphism and an increase of white matter hyperintensity lesioned areas observed by MRI in AD patients [313, 314]. WAMs present higher expression levels of ubiquitin-specific protease 18 (Usp-18), which contributes to microglial quiescence by negatively regulating the activation of Stat-1 and abrogating IFN signaling [311, 315]. WAMs also rely on TREM2 signaling and are age-dependent. In AD mouse models, WAMs signature is *APOE*-dependent, whereas in aged brains, WAMs show an *APOE*-independent signature [311]. As previously mentioned, the study from He and colleagues related to the apoE receptor LRP1 indicated that LRP1 might modulate microglial response via the JNK and NFκB pathways [297]. Since white matter injury is related to myelin and axonal loss after different events, additional studies point out that selective blockade of LRP1 in OPCs could potentially enhance myelin repair [316, 317]. Conversely, in a rat model of subarachnoid hemorrhage, an intraperitoneal dose of an apoE-mimic peptide and LRP1 ligand (COG1410) revealed that microglial LRP1 activation alleviates white matter injury and improves neurological functioning by shifting microglia towards an M2 phenotype through the activation of the Shc/PI3k/Akt signaling pathway [318]. A study from Hammond and Colleagues evaluated the microglial subpopulations from embryonic stages until old age and after the induction of a local demyelinating injury. Among the microglial subpopulations found, the authors performed a comparison between axonal-tract-associated microglia (ATM), DAM, and injury-responsive microglia (IRM) by using canonical correlation analyses. The authors observed that ATM microglia are characterized by upregulated developmental genes, such as *Gpnmb* and *Fabp5,* and IRM microglia presents downregulated expression levels of *P2ry12* and *Cx3cr* and upregulated transcription factor *Ifi204,* interferon-response genes *Ifi27l2a* and *Cxcl10* [267, 308]. Interestingly, ATM, DAM, and IRM microglial groups shared 12 core genes among their transcriptomic structure, including among those genes *Spp1*, *Lpl,* and *apoE* [319]. Sala-Frigerio and colleagues evaluated more than 10 thousand individual microglial cells derived from the cortex and hippocampus of APP mice. The authors observed multiple microglial clusters; among them, transiting-response microglia (TRM) and Activated-response microglia (ARM) transcriptomic profiles were similar. These clusters present an increased expression of apoE and inflammatory markers paired with the downregulation of homeostatic microglial markers. ARMs also present an overexpression of MHC-II genes. TRM express relatively lower apoE and MHC-II markers than ARMs and do not appear to show tissue regeneration genes [308]. They also revealed ARMs as the meeting point for aging processes, sex, and AD risk factors because ARM response was abolished with *APOE* deletion. Finally, the authors also identified a small microglial sub-cluster corresponding up to 1,2% of the microglial subpopulations called cycling/proliferating microglia (CPM), enriched in genes related to DNA replication, chromatin rearrangement, and the cell cycle, such as *Top2a*, *Mcm2*, *Tubb5*, *Cdk1,* and *Mki67*.

Aging is also a significant factor influencing microglial function. Marschallinguer and colleagues performed a study on C57BL/6 J male mice to assess structural differences between young and old microglia (three-month versus twenty-month-old mice) by analyzing their cytoplasmic content through transmission electron microscopy. The authors identified a novel state of microglial cells present in the aging brain called lipid-droplet accumulating microglia (LDAM). LDAM were abundant in the aged hippocampus and rich in neutral lipids, such as tri-, di- and monoacylglycerols and cholesteryl esters. Transcriptional analysis of LDAM revealed a differential expression of genes related to the production of ROS and NO (*kl, ppp1cb, jak, cat, rap1b*), as well as genes related to phagosome maturation pathways, vesicular transport (*rab5b* and *rab7*), and *cd22*, which negatively regulates microglial phagocytosis. The authors also compared the LDAM transcriptional phenotype to the previously characterized microglial phenotypes, such as aging microglia, DAM, MGnD microglia, and the clusters discovered by Li and colleagues, and Hammond and colleagues. They determined that the LDAM transcriptional phenotype was distinct to those previously characterized, standing out by presenting defective phagocytosis, together with a high production of reactive oxygen and nitrogen species and pro-inflammatory cytokines. The authors also investigated the plausible genetic regulators of microglial lipid droplet formation by CRISPR-Cas9 screening in a microglial mouse-derived BV2 cells, finding *slc33a1*, *snx17*, *grn*, and *vps35* as neurodegeneration-related genes and thus pointing to a possible link between LDAM and neurodegeneration. Nonetheless, it is interesting that neither *APOE* nor TREM2 was regulated in LDAM [320]. Thus, more research must be guided towards the study of microglial heterogenicity, as well as determining how factors such as age, sex, and the *APOE* genotype impact microglial phenotypes and their function during health and disease. The microglial phenotypes described and the most relevant hallmark genes expressed are summarized in Table 4.

**Table 4:** Some of the Microglial phenotypes identified by RNA-seq techniques up-to-date

Microglial Phenotype	Hallmark genes	References
DAM	↓ Homeostatic genes (P2ry12, Cx3cr1, Tmem119) ↑ **ApoE**, Itgax Ctsd, Igf1, Lpl, Clec7a, Tyrobp, Trem2	[267]
MGnD	↓ Homeostatic genes (P2ry12, Tmem119, Gpr34, Csf1r, Hexb, and Mertk) ↑ **ApoE**, Spp1, Itgax, Lgals3, Axl, Clec7a, and Ccl2	[269]
WAM	↓ Homeostatic genes (P2ry12, P2ry13, Csfr1r, Cx3cr1, Hexb, and Tmem119) ↑DAM genes (**ApoE**, Cst7, Bm2, Lyz2, Cd63, Clec7a), Cathepsins (tsb, Ctss, and Ctsz), MHC-II genes (H2-D1 and H2-K1)	[311]
ATM	↑**ApoE**, developmental genes (*Gpnmb* and *Fabp5*), *Spp1*, *Lpl Gpnmb*, *Igf1*, *CD68*, immunomodulators (*Lgals1*, *Lgals3*), *lamp1*, *cd68*	[319]
IRM	↓ P2ry12 and Cx3cr1, ↑ **ApoE**, cell proliferation markers (Birc5), interferon and immune response (Cxcl10, Ifit2, Ifit3, Ifitm3 Oasl2, and Irf7), chemoattractant genes (CCl4)	[319]
TRM	↓ P2ry12 ↑ **ApoE (**lower levels than ARM)**,** inflammation markers (Cst7)	[308]
ARM	↓ P2ry12 ↑ **ApoE,** MHC-II genes (H2-Aa, H2-Ab1, and Cd74), tissue repair genes (Spp1, Gpnmb, and Dkk2), AD-related genes (Ctsb, Bin1, and Pld3)	[308]
CPM	↑ Genes DNA replication, chromatin rearrangement, and the cell cycle (Top2a, Mcm2, Tubb5, Cdk1, and Mki67)	[308]
LDAM	↑ Genes related to the production of ROS and NO (KL, PPP1CB, JAK, CAT, RAP1B), lipid-related genes (PLIN3), lipogenesis (ACLY)	[320]

↓ (downregulated), ↑ (upregulated), *DAM* Disease-associated microglia, *MGnD* Microglial neurodegenerative phenotype, *WAM* White-matter-associated microglia, *ATM* Axonal-tract-associated microglia, *IRM* Injury responsive microglia, *TRM* Transiting response microglia, *ARM* Activated response microglia, *CPM* Cycling/proliferating microglia, *LDAM* Lipid-droplet a-associated microglia

### *APOE* and astrocytic function

Although apoE can be produced by different cell types in the brain, the vast majority of brain apoE is generated by astrocytes [81, 321]. Astrocytes have a supportive role in neuronal function, mature neurons predominantly rely on cholesterol provided by astrocytes, and apoE plays an essential role in transporting cholesterol from astrocytes to neurons [322]. Human iPSC in vitro models have evidenced that apoE protein and mRNA levels are decreased in *APOE4* astrocytes compared to *APOE3* astrocytes [178]. Astrocyte-derived apoE4 is poorly lipidated compared to apoE3, suggesting that astrocyte-derived apoE3 is more efficient in transporting cholesterol to neurons than apoE4 [323, 324].

Although it remains unclear why apoE4 particles are less lipidated than apoE3, the poor lipidation status of apoE4 is likely linked to impaired cholesterol metabolism in astrocytes. The study by Julia TCW and colleagues showed an increased cholesterol synthesis by *APOE4* compared to *APOE3* IPSC-derived astrocytes, a finding in line with the previously described increased cholesterol biosynthesis, including increased intracellular cholesterol levels and secretion, by *APOE4* IPSC-induced astrocytes [325]. Lin and colleagues also described increased intracellular cholesterol accumulation in *APOE4* compared to *APOE3* IPSC-derived astrocytes [178]. Of note, increased cholesterol secretion by astrocytes is contradictory to previous findings in *APOE* knock-in mice, as cholesterol release was observed to be increased in astrocytes from *APOE3* knock-in compared to *APOE4* knock-in mouse astrocytes [189, 323]. Moreover, genetic expression of *APOE4* in iPSC-derived astrocytes will also induce an increase in the intracellular unsaturated fatty acid levels and lipid droplets, which it is possible to restore with choline supplementation [326]

Interestingly, although lipid metabolism regulated by astrocytes in the brain mainly includes lipid transport from astrocytes to neurons, recent studies have described a ‘neuron-astrocyte shuttle’, where toxic fatty acids in neurons are transferred to astrocytes for neutralization, a transfer that also relies on apoE [327–329]. Neurons have a limited capacity to store fatty acids in lipid droplets or to catabolize fatty acids [330]. Excessive free fatty acids in the cytosol could lead to lipid peroxidation, toxicity, mitochondrial dysfunction, and neurodegeneration. Therefore, it is highly relevant to control fatty acid metabolism [331–333]. In contrast to neurons, astrocytes could store fatty acids in lipid droplets and catabolize the lipids by mitochondrial respiration [327, 334]. The transport of fatty acids from neurons to astrocytes to neutralize or avoid fatty acid-induced toxicity depends on apoE [327]. More specifically, *apoE*4 has shown to be less efficient in transporting fatty acids to astrocytes [328]. In addition, the same study also described increased lipid droplet accumulation and reduced fatty acid oxidation in astrocytes expressing *APOE4*, causing not only diminished transfer of toxic fatty acids away from neurons but also reduced capacity of astrocytes to neutralize toxic lipid species [326, 328].

In addition to the role of astrocytes in lipid metabolism in the brain, astrocytes also have a supportive role in brain energy homeostasis. Humanized *APOE4* knock-in astrocytes have a reduced mitochondrial capacity compared to *APOE3* knock-in astrocytes [328, 335]. In addition, *APOE4* astrocytes are linked to altered glucose utilization, e.g., increased glycolytic activity, reduced glucose uptake and oxidation, increased aerobic glycolysis and increased lactate synthesis [336, 337]. *APOE4* astrocytes appear to present a shift in glucose metabolism toward a less oxidative tricarboxylic acid cycle and an increased glucose flux towards the pentose phosphate pathway and de novo- biosynthetic routes [336]. Qi and colleagues argued that mitochondrial respiration preferentially relies on glucose metabolism rather than fatty acid β-oxidation, e.g., because of a higher oxygen consumption rate in *APOE4* in the presence of glucose substrate [328]. Other authors have reviewed additional mechanisms through which the *APOE4* polymorphism impacts glial cell functioning [38]. Further research is required to understand the bioenergetic function in *APOE4* astrocytes thoroughly.

## *APOE* in Alzheimer’s disease

### *APOE*, amyloid-β load and AD pathology

In 1993, the link between apoE4 and LOAD was first described by Corder and colleagues and thereafter the number of studies on *APOE4*, LOAD and Aβ pathology increased tremendously [338]. In general, *APOE4* carriers have an increased plaque load and density compared to non-*APOE4* carriers [160, 339, 340]. Similarly, mice expressing the human apoE4 isoform have more Aβ deposits and neuritic amyloid plaques than mice expressing other human apoE isoforms [66, 341]. The effect of human *APOE* on Aβ deposition is isoform-dependent with *APOE2*, opposite to *APOE4*, showing the lowest Aβ plaque deposition [66]. A complete lack of *APOE* expression in AD transgenic mice overexpressing human mutant APP, but not PSEN1, dramatically reduces deposition of Aβ in the brain and lowers both Aβ_40_ and Aβ_42_ deposits, highlighting the important role of human *APOE* in amyloid deposition [341–343]. Nonetheless, *APOE* deletion in a more aggressive AD mouse model containing mutations in both APP and PSEN1, such as APP/PS1 mice, induces an overall increase in plaque size [236]. Interestingly, the prior study also observed a decrease in the Aβ plaque load positive for X-34, an amyloid stain that preferentially binds mature fibrillar plaques.

The effects of *APOE* deletion in mouse models used to study amyloid pathology are not fully understood and therefore require further investigation.

Compared to endogenous *m-apoe* expression, all human *APOE* isoforms reduce plaque deposition and onset, with the strongest reduction induced by expression of the human *APOE2* isoform in mice [66, 344]. Besides parenchymal amyloid plaque deposition, *APOE4* has also been linked to cerebral amyloid angiopathy (CAA) formation in an AD transgenic mouse model (Tg2576) cross-bred with humanized *APOE*-knock in mice [344].

More recently, amyloid positivity in the brain detected by positron emission tomography (PET) scan using Pittsburgh compound B (PiB) and reduction in CSF Aβ_42_ were shown to be predictors of clinical AD [345]. *APOE* genotype was shown to affect amyloid PET scan results and CSF Aβ levels, with cognitively normal *APOE4* carriers evidencing increased PIB^+^ amyloid burden in the brain and reduced levels of Aβ_42_ in CSF compared to non-*APOE4* carriers [346–348]. The strongest effect with age on amyloid burden and Aβ_42_ CSF levels was seen in *APOE4* homozygotes, suggesting a dose-dependent *APOE4* effect on amyloid plaque burden prior to clinical AD onset [346].

Although the mechanistic link between *APOE4* and plaque development remains poorly understood, a critical role of *APOE4* in plaque seeding has been described [290, 349]. Antisense oligonucleotide (ASOs) that suppress *APOE* expression prior to plaque onset reduced plaque load in humanized *APOE4* knock-in / APP/PS1-21 mice compared to non-ASOs treated controls [290]. Interestingly, *APOE* reduction by ASOs after plaque onset did not affect plaque load in *APOE4* / APP/PS21 mice. Additionally, another study also supports a role of *APOE4* predominantly in plaque seeding and not in plaque growth [349]. In this study, inducible mouse models were generated overexpressing human *APOE3* or *APOE4*. Overexpressing human apoE4 during the first 6 months of age, prior to plaque onset, strongly increased plaque load, Aβ oligomers and Aβ levels compared to mice not expressing human apoE. Overexpression of human apoE4 from 6 to 9 months of age, thereby starting apoE expression directly after plaque onset, did not influence plaque load and Aβ levels, strongly indicating an apoE4-dependent effect on plaque seeding, but not growth. Human apoE3 is suggested to have a weaker effect on plaque formation as both these two studies did not find a clear effect in the presence of apoE3 [290, 349].

The exact mechanism promoting amyloid plaque formation remains poorly understood, but is most likely caused by either increased Aβ production and/or aggregation, and/or decreased Aβ clearance. Although some conflicting data have been published [350], most studies found that human apoE, in particular apoE4, enhances Aβ fibril formation in vitro [291, 351, 352]. Differences in the preparations of apoE and/or Aβ peptides could be an explanation for the observed differences in in vitro Aβ fibrillization studies. The lipidation status of apoE, especially reduction of apoE lipidation by depleting ABCA1, contributes to increased amyloid deposition in AD transgenic mice [353, 354]. ApoE co-deposits within amyloid plaques [355] and the form of apoE localizing to insoluble Aβ deposits in AD appears to be poorly lipidated [354].

Prior to the onset of amyloid plaques, Aβ accumulates intracellularly in AD vulnerable neurons [356]. Intraneuronal accumulation of Aβ_1-42_ in late endosomal and lysosomal compartments is enhanced by the presence of apoE4 in mice [357]. The binding of lipidated apoE to ApoER2 receptors could trigger APP and BACE1 internalization, resulting in increased intracellular Aβ generation [358]. ApoE4 binding leads to more enhanced APP and BACE1 endocytosis than apoE3 and apoE2, consequently resulting in a stronger increase in Aβ production by apoE4. More recently, an apoE-isoform dependent effect on APP transcription and Aβ secretion was reported, with apoE4 inducing the strongest increase in APP transcription, followed by apoE3 and lastly apoE2 [359].

Besides increased Aβ generation and aggregation, amyloid pathology is also influenced by disrupted Aβ clearance. Aβ can be cleared from the brain via several clearance pathways, including enzymatic degradation, clearance through the BBB, and cellular internalization and subsequent lysosomal degradation by astrocytes, microglia and neurons [34, 360]. In general, apoE4 is associated with lower Aβ clearance [34, 361]. However, the exact mechanisms of Aβ clearance and the role of apoE in this are complex. One of the mechanisms to clear Aβ from the brain is by proteases, such as neprilysin and insulin-degrading enzyme (IDE) [362, 363]. Both intracellular and extracellular enzymatic clearance of soluble Aβ is enhanced by apoE [364]. Highly lipidated apoE increases Aβ clearance by proteases to the highest extent [364]. Human post-mortem studies have shown reduced neprilysin and IDE expression in *APOE4* compared to non-*APOE4* carriers, suggesting potential differences among apoE isoforms on proteolytic activity [365, 366].

Aβ can be degraded intracellularly via lysosomal degradation in astrocytes, microglia, and neurons. In all cell types, apoE4 has been linked to a reduced cellular clearance of Aβ [364, 367, 368]. ApoE influences Aβ internalization and degradation in microglia in an isoform-dependent manner with the slowest Aβ uptake and the least efficient Aβ degradation by apoE4 [178, 364]. ApoE was also reported to moderate Aβ cellular degradation by reducing intracellular cholesterol levels, without direct interaction between apoE and Aβ being required [369]. Reducing intracellular cholesterol in microglia results in accelerated endosomal transport of Aβ towards lysosomes [369]. In astrocytes, *APOE4* induces impaired Aβ_1-42_ internalization compared to *APOE3* astrocytes [178]. An *APOE*-dependent mechanism of Aβ clearance in astrocytes was proposed as *APOE*^*−/−*^ astrocytes were unable to degrade Aβ [368]. However, more recently, Aβ uptake and clearance by LDLR in astrocytes was shown to also take place in the absence of *APOE*, pointing towards an *APOE*-independent Aβ uptake mechanism [370]. An indirect apoE-induced inhibition of Aβ uptake by competing for the same receptors, such as LRP1 receptors, could block Aβ internalization and subsequent degradation [371]. Neurons can also degrade Aβ by cellular internalization and lysosomal degradation [367]. Although the research on neuronal Aβ clearance and apoE remains limited, apoE3 seems to enhance neuronal Aβ uptake, endosomal trafficking and lysosomal degradation more strongly than apoE4 [367].

Another clearance mechanism of Aβ is by the BBB. Both in vitro and in vivo studies showed that apoE could modulate BBB-related Aβ clearance [372–374]. ApoE4 impaired Aβ removal via the BBB compared to apoE3 and apoE2. The presence of apoE4 has also been associated with BBB breakdown [375–377]. Impairment of the BBB might further dysregulate Aβ clearance from the brain. The precise mechanism of apoE in Aβ clearance by the BBB remains poorly understood. ApoE2, apoE3, apoE2-Aβ and apoE3-Aβ complexes can be cleared by LRP1 and VLDLR at the BBB at a substantial faster rate than apoE4 and apoE4-Aβ complexes [372]. Additionally to BBB clearance of Aβ, apoE also modulates soluble Aβ clearance from interstitial fluid (ISF) in mice in an apoE-isoform dependent manner, shown by an increased half-life of Aβ in ISF in PDAPP/*APOE4* compared to PDAPP/*APOE3* and PDAPP/*APOE2* mice [378]. More research is needed to better understand the role of apoE in Aβ clearance from the ISF.

In sum, *APOE4* has been highly associated with changes in amyloid pathology in AD. *APOE4* has shown to increase plaque load, in particularly by enhancing plaque seeding rather than growth, and could influence Aβ production and aggregation. At the same time, Aβ clearance has been shown to be least efficient in the presence of an *APOE4* genotype. The most crucial process of *APOE* related to amyloid pathology remains unclear, and additional studies are required to increase the knowledge about the effect of *APOE* and amyloid pathology in AD.

### *APOE* and Tau pathology

As priorly mentioned in our manuscript, aggregates of hyperphosphorylated tau protein conforming NFTs constitute one of the hallmarks of AD. NFTs correlate with the cognitive deficits observed in AD patients, but whether they drive AD pathology is still under debate [379–381].

Initial histopathological evidence established a relation between apoE protein and NFTs in AD brains [382–384]. Tau pathology is tightly related to neurodegeneration, and it has long been considered that tau aggregation into NFTs result in a toxic gain of function [385]. Tau pathology originates in the EC and builds out to the hippocampus, temporal and cortical areas, been the cerebellum the least affected area. Even so, the differential vulnerability of brain regions to tau pathology is still unknown [386]. Throughout different studies, *APOE4* carriers display a robust amnestic phenotype with a more severe and typical medial and temporal spread of tau, following the stereotypical Braak staging system characterized in AD [387–390]. On the other hand, *APOE4* non-carriers show a non-Braak pattern of tau PET uptake with lower levels in the EC and higher in the neocortex, a finding that is hypothesized to be mediated by Aβ deposition [391–393].

There are numerous theories for p-tau spreading through the brain. Various studies point out that it is a process driven by microglia, whereas other authors also suggest tau spreading occurs through neuronal communication pathways [394, 395]. Van der Kant and colleagues reviewed the role of microglia in AD-related tau pathology independently of Aβ, being apoE a key regulator of this phenomenon [396].

On that note, the *APOE4* genotype exacerbates tau-mediated pathogenesis, neurotoxicity neuroinflammation, and neurodegeneration in numerous pre-clinical models [390, 397–400]. The study from Kang Su and colleagues observed that apoE4 potentiates tau neurotoxicity by binding the monoamine transporter 2 (VMAT2), inhibiting neurotransmitter active transport into synaptic vesicles, and eventually leading to Locus Coeruleus (LC) degeneration [400]. In line with the previous findings, in the P301S mouse model of tauopathy, together with knock-in or knockout *APOE*, *APOE4* expressing mice showed the strongest tau-induced neurodegeneration compared to the other two isoforms, whereas *APOE* knockout mice were protected from this effect [397]. This same research group from David M Holtzman recently showed the potential of reducing *APOE4* levels with antisense oligonucleotides in P301S/*APOE4* mice, which protected against tau pathology [401]. For a review on how *APOE* affects both tau pathology and neurodegeneration through its immunomodulatory function, the reader is referred to the review article from Shi and Holtzman [36].

Data from human iPSCs-derived cell types have shown a relation between apoE4 and tau pathology in neurons and glial cells [178, 402, 403]. It has been documented that selective removal of astrocyte-derived apoE4 protects against tau-mediated neurodegeneration [404]. AAV-mediated overexpression of *APOE* human isoforms in astrocytes revealed that the astrocytic overexpression of *APOE4* enhances tau phosphorylation and aggregation, both in vitro and in the P301L mouse tau model [399]. However, AAV-mediated neuronal overexpression in rTg4510 and PS19 mice failed to alter p-tau burden [405]. Additionally, other tauopathy models have addressed an association between the cleaved c-terminal fragment of apoE4 and tau [383, 406].

In terms of brain imaging evidence, studies using the tau PET tracer ^18^F-AV-1451, also known as ^18^F-flortaucipir, showed contradictory results, ranging from an association between medial temporal tau and apoE4 independently of Aβ in cognitive performance [407]; to establishing no association between medial temporal tau and apoE4 in cognitively normal individuals, mild cognitive impairment (MCI) or AD patients [408, 409]. The use of the tau tracers ^18^F-MK6240 and ^18^F-flortaucipir revealed a relationship between medial temporal tau and apoE4 beyond Aβ [390, 410]. Rodriguez-Vieitez and Nielsen thoroughly reviewed the effects of total tau load in this matter [411]. On top of that, an association between *APOE4* and sex has been described in PET studies, with women carrying the allele showing a more widespread tau pathology [412, 413].

Analysis of CSF from AD patients and controls revealed that both apoE3 and apoE4 are positively associated with total tau and phosphorylated tau concentrations [199]. Similar findings have been reported from MCI and AD groups, but only concerning *APOE4* carriers, and when comparing tau and apoE levels [414–417]. Another study showed that in CSF of *APOE4*-carrying AD patients, tau levels were associated with impaired cortical plasticity, cognitive decline, and astrocyte survival [418]. Overall, correlating apoE and tau levels in CSF samples is complex and not as evident as relating apoE to Aβ levels. Most findings are contradictory and are highly dependent on the disease stage [411]. It is also relevant to consider that measuring CSF tau only provides information on global soluble tau in the entire brain, while tau PET imaging informs of the specific regional distribution of NFTs [409]. In addition, tau PET seems to be a more accurate reflection of disease severity than CSF studies, and concordance between PET and CSF tau levels may rely on the disease stage and which tau fragments are studied [419, 420].

Interestingly, the protective effect of *APOE2* in Aβ load has not been reported concerning tau pathology since there are no significant changes in the levels of CSF tau in these carriers, with one exception reporting lower phosphorylated tau levels [421, 422]. Tau PET studies reported similar findings, evidencing that the protective effect of *APOE2* on developing AD is primarily linked to the resistance against Aβ deposition [409]. Nevertheless, *APOE2* homozygosity has been shown to enhance tau pathology, increase the risk of primary tauopathies, and is more frequent in primary age-related tauopathy as opposed to AD cases [423, 424].

Despite this data, direct interaction between apoE and tau has not been reported in vivo, and it has to be taken into account that apoE is primarily secreted, whereas tau tends to be located in intraneuronal and axonal regions, although evidence has shown that it can also be present in the extracellular space [425]. The mechanism by which the *APOE* genotype might affect tau deposition is unknown, but the two primary metabolic receptors in charge of mediating clearance of apoE lipoproteins, LDLR and LRP1, have been shown to regulate tau propagation in opposite ways. LDLR overexpression reduces tau pathology via apoE-linked mechanisms, whereas LRP1 knockdown reduces tau propagation [426, 427]. No differential effect has been established in terms of apoE isoforms, but Rauch and colleagues suggest that apoE4 would be the least effective at inhibiting tau spread, since as previously commented, this genotype presents the lowest levels of circulating apoE protein in the brain.

To conclude, there is still much debate about the influence of *APOE* genotypes, particularly on how *APOE4* impacts tau burden. In the context of AD, it is difficult to isolate a direct connection between apoE and tau due to the effect of Aβ, and it is argued that the effect of the *APOE4* allele on tau pathology may be secondary to the marked effect of the *APOE* genotype on Aβ burden.

### *APOE* and synaptic function in AD

Synapses are among the earliest cellular sites affected by AD, and a loss of synaptic terminals appears to be the best correlate to cognitive impairment in AD patients [428–431]. Reduced levels of pre-and post-synaptic proteins, such as synaptophysin and neurogranin, are found in AD post-mortem brains compared to age-matched controls [432, 433]. Brain regions with a higher active default state, e.g., the hippocampus and EC, seem particularly vulnerable to AD [434]. Aβ oligomers, rather than amyloid plaques, have been shown to cause synaptic toxicity, resulting in synaptic dysfunction, loss of synapses, and disrupted synaptic plasticity, including long-term potentiation (LTP) and long-term depression (LTD) [435–438]. Oligomeric Aβ localized near plaques is detected at post-synaptic terminals and correlates with spine loss close to amyloid plaques [435]. Aβ_1-42_ accumulation occurs at synaptic terminals even earlier than amyloid plaques formation in AD transgenic mice suggesting an early effect of Aβ on synaptic pathology [439, 440].

*APOE4* is among the LOAD risk genes related to synaptic function impairments in AD [441]. ApoE, as the primary lipid carrier in the brain, will transport lipids, e.g., cholesterol, from astrocytes to neurons. Cholesterol is of high relevance for axonal growth, synapse formation, and spine remodeling; highlighting the role of apoE as a cholesterol transporter in the maintenance of synapses [442–444]. Notably, during neuronal stress and injury, apoE expression and cholesterol transport from astrocytes to neurons increase as part of a neuronal repair mechanism [445]. Moreover, astrocytic apoE may modulate neuronal function by altering histone acetylation in an isoform-specific manner (apoE3 > apoE4), and astrocytic-derived microRNAs (miRNAs) may selectively silence genes related to neuronal cholesterol synthesis [446]. Even without neuronal injury, apoE has been associated with changes in neuronal outgrowth and synapse density in normal brain functioning [234, 447]. Prior studies have reported generalized reduced neuronal outgrowth in *APOE4* than in *APOE3* neurons [447–450]. Additionally, synaptic density is decreased in neurons expressing human apoE4 [234], perhaps by a reduced spine formation or an enhanced spine removal, as shown in in vitro models [451].

AD is also related to disrupted synaptic plasticity. Multiple studies previously investigated the role of apoE in synaptic plasticity, particularly on LTP. Overall, the *APOE4* genotype is associated with decreased LTP compared to *APOE3* in mouse and in vitro models [452, 453]. Reduced LTP induction induced by apoE4 is dependent on NMDA receptor functioning [454]. Although most studies support a detrimental effect of apoE4 on LTP, conflicting data have reported increased LTP in young *APOE4* mice compared to *APOE3* mice [455]. The differences in LTP stimulation protocols, such as high-frequency stimulation, or tetanic stimulation, could potentially explain the different effects of apoE4 observed on LTP [106]. In line with reduced LTP in the presence of apoE4, poor learning and memory outcomes have been described in *APOE4* targeted-replacement mice, suggesting that *APOE4* impairs synaptic function and also worsens cognitive function [456–458].

Whether apoE affects synaptic function by its direct presence at synaptic terminals remains poorly studied. ApoE is present in a subset of synapses, co-localizing to 18–36% of synapses [459]. A recent study observed a synapse-associated localization of apoE in primary neuron cultures, in which both apoE3 and apoE4 co-localize with vGlut1-positive pre-synaptic terminals [460]. ApoE co-localizes with synaptic markers in both healthy controls and AD subjects, pointing towards a not purely pathological role of apoE at synaptic terminals. Interestingly, enhanced accumulation of oligomeric Aβ is observed in synapses positive for apoE [459]. In addition, apoE/Aβ complexes accumulate in human cortical synaptic terminals, especially during AD, and their expression increases in the presence of apoE4, compared to brains from non-*APOE4* carriers [461]. Nonetheless, additional research is still needed to address whether ApoE4-induced synaptic dysfunction is dependent or independent of Aβ/APP accumulation.

Although many apoE-related changes in synaptic function have been described in observational studies, the mechanisms by which apoE affects synaptic function remain not fully understood. For instance, in vitro electrophysiological evaluation in vitro of human hiPSCs neurons treated with astrocyte-derived extracellular vesicles (EVs) revealed an enhanced electrophysiological function paired with an anti-apoptotic behavior. Among the cargos found inside the EVs of this study, heat shock proteins, LRP1, alpha-synuclein, and apoE were detected, among others. [462]. However, the previous study did not evaluate the effect of the different apoE isoforms, which would be of great interest in the future. In line with the prior findings related to astrocytic apoE, the Holzman group observed that astrocytic deletion of *APOE4* rescues synapse loss [398]. Another study using male embryonic stem cells observed an effect of apoE on synaptogenesis and concluded that apoE stimulates CREB and cFOS signal transduction cascades and isoform-dependent decrease of neuronal signaling (ApoE2 < ApoE3 < ApoE4) [184].

Novel approaches such as synaptometry time of flight (SynTOF), a technique combining mass spectrometry and flow cytometry, have been developed to elucidate cerebral single synaptic events in health and disease. Montine TJ and colleagues have implemented the SynTOF technique in synaptosomes derived from non-human primates, PS/APP mice, and post-mortem tissue from individuals solely with AD neuropathologic change [463]. Some of the most striking findings in this study included the absence of Aβ at human synapses, diverging from prior authors’ findings (e.g. [459]. The authors also observed an increase in the hippocampal synaptic levels of the marker CD47, apoE, and a possible link between low apoE levels in pre-synaptic events and AD resilience [463]. Although one of the possible limitations of this technique is relying on optimal antibody binding, it constitutes a promising tool for future studies.

Impairment of endosome recycling has been described as a potential mechanism causing synaptic dysfunction induced by apoE4 [452, 464]. *APOE4* has been associated with significantly reduced endosomal recycling and increased intracellular accumulation of apoE [130, 452]. ApoE4-induced endosome recycling impairment could result in the trapping of apoE, ApoER2 receptors, and glutamatergic receptors involved in synaptic plasticity, such as NMDA and AMPA receptors in endosomes [452]. As a consequence, synaptic receptor trapping in endosomes induces impaired synaptic regulation. Additionally, apoE4-induced intracellular trapping of ApoER2 receptors results in reduced ApoER2 receptor binding by Reelin [465], leading to dysfunctional synaptic plasticity regulation. A recent study discovered the involvement of NHE6, a proton channel present in early endosomes; since NHE6 inhibition rescued impaired Reelin-mediated synaptic plasticity induced by apoE4 [464]. More research is required to define better the role of apoE and its isoforms on synaptic function.

### Implications of *APOE* in autophagy

Endocytosis, phagocytosis, and autophagy are closely related functions involved in the cellular trafficking and recycling system. Briefly, endocytosis is involved in the recycling and internalization of macromolecules from the plasma membrane, autophagy in the removal of intracellular sources and organelles, and phagocytosis in the degradation of extracellular materials. Importantly, autophagy and phagocytosis share many molecular pathways and mediators as LC3, Beclin1, ATG5, or ATG7 in microglia. [466–468]. Regarding autophagy, although there are three forms (microautophagy, macroautophagy, and chaperone-mediated autophagy), the term typically refers to macroautophagy. In addition to the well-known response to starvation, autophagy is also involved in different processes as degrading dysfunctional organelles, pathogens, or aggregated proteins [469, 470].

The specific role of autophagy in AD has been extensively investigated in the last years (see review [469]). Already in the early preclinical AD stages, there are alterations in the autophagy-lysosomal network associated with the intracellular accumulation of Aβ [471, 472]. Endo-lysosomal trafficking and autophagy are essential in the formation of Aβ from APP [471], APP degradation and Aβ clearance [466, 473]. Autophagy is an additional mechanism for the clearance of Aβ by microglia [474]. A failure in the complex pathway of cell trafficking and autophagy mediates Aβ accumulation [475]. Furthermore, this alteration influences other elements involved in AD pathophysiology, such as neuroinflammation [476].

Autophagy and neuroinflammation interact at several levels regulating each other bidirectionally. Several cytokines induce or inhibit autophagy; meanwhile, autophagy decreases the secretion of other cytokines [477]. However, this relationship is much more complex in the context of AD; as previously described above, an alteration in autophagy is associated with Aβ accumulation. Therefore it has been observed that autophagy in microglia reduces neuroinflammation [478], but the secretion of pro-inflammatory factors is associated with an increase in autophagy [479, 480]. All this indicates that other factors, such as differential gene expression, must be involved in this complex interplay.

Some LOAD-associated alleles are linked with endocytic/autophagic pathways, being *APOE4* the most relevant [481, 482]. ApoE is involved in Aβ internalization by binding to the Aβ_12-28_ region, being apoE4 much less efficient in this function compared to apoE2 and 3, leading to a higher accumulation of Aβ in both patients and animal models carrying *APOE4* [471, 483, 484]. Nevertheless, the specific mechanism by which *APOE4* alters autophagy is not yet fully understood, although several publications support that it is one of the main pathological processes regulated by *APOE4* in AD (see review [485]).

Concerning *APOE4* effects upon glial cell autophagy, Simonovitch and colleagues observed in vitro that *APOE4* expression in astrocytes induced lower autophagic flux and a reduced capacity to clear Aβ compared with *APOE3*-astrocytes [470]. Then, in vivo, they noticed a decrease in autophagy and mitophagy in the hippocampus of *APOE4* mice [486]. Also in *APOE4* mice, Nuriel and colleagues observed, using transcriptomics, a dysregulation of the endosomal-lysosomal pathway compared to *APOE3* mice [487]. Likewise, Pomillo and colleagues presented in vitro and in vivo evidence of microglial autophagy impairment and defective Aβ degradation as AD progresses [488].

Regarding human brain samples, Parcon and colleagues observed a reduction in mRNA transcripts related to autophagy in *APOE4* carriers compared with *APOE3*. They noticed lower mRNA levels of LC3, p62, or LAMP2, all of which are essential components in the autophagic pathway. In the same work, authors observed, using *APOE3*-expressing astrocytes (T98G), an upregulation on mRNA levels of LC3, p62, and LAMP2 after autophagy induction. This response was not detected in *APOE4*-T98G cells [489]. Previously, Zhu and colleagues noticed an apoE4-specific increase in the expression of synaptojanin 1 (SYNJ1), a phospholipid regulator which, when induced, caused an increased Aβ clearance and reduced synaptic damage in an AD mice model [490, 491]. Consequently, there is an alteration of autophagy in *APOE4*-models (in vitro and in vivo) and in human *APOE4*-carriers. Furthermore, this alteration is part of the vicious circle associated with the progression of the pathology since it involves other elements such as neuroinflammation, cell death, among others.

The complete mechanism that underlies the link between *APOE4* and autophagy is not yet fully understood, but some processes have been suggested to be involved, such as the interaction with autophagy regulators (i.e., TFEB and mTOR) or phospholipid dysregulation. Parcon and colleagues demonstrated in vitro and in silico that apoE4 could bind to the CLEAR promoter, blocking or interfering with the CLEAR-binding of the transcription factor EB (TFEB) [489], a master regulator of lysosome function and autophagy [492]. Subsequently, Lima and colleagues found that the interaction with CLEAR was apoE4-specific, as they did not observe interaction with apoE3 nor apoE2 [493]. Reduced levels of TFEB in lymphocytes and monocytes have been detected in patients with AD, so as in mouse models [494, 495]. Therefore, according to these works, *APOE4* could reduce the autophagic response through interference with TFEB activation.

mTORC1 is one of the primary regulators of cell growth with multiple pleiotropic functions, such as the repression of autophagy [496]. In AD, the mTORC1 pathway is upregulated, correlating with cognitive decline in AD patients [497, 498]. Although several works have studied the relation between *APOE4* and the mTORC1 pathway, to date, there is no consensus on this issue. Even though apoE4 receptors VLDLR and apoER2 activate PI3K/AKT/mTORC1 [499, 500], Yates and colleagues observed that the alteration in the mTORC1 pathway was independent of apoE status in brains of AD-patients [501]. However, Li and colleagues noticed in the hippocampus of *APOE4* Wistar rats an activation of the mTORC1 pathway, decreased autophagy, and AD-like alterations [502]. Finally, different studies showed that treatment with rapamycin (inhibitor of mTOR) has a protective effect in the brain of *APOE4* transgenic mice and E4FAD mice (5xFAD cross-bred with *APOE4* mice) [503–505]. These and other previous results have raised interest in rapamycin as a drug of potential interest in AD treatment [506].

The control of autophagy by *APOE* could involve other mechanisms such as the dysregulation of phospholipids (e.g., phosphoinositol phosphate (PIP) and phosphoinositol biphosphate (PIP2)). PIP and PIP2 mediate autophagy, regulating the mTOR pathway or controlling membrane dynamics [507]. Zhu and colleagues observed an exacerbated reduction in PIP2 in *APOE4* carriers’ brains compared to *APOE3*. Decreased levels of PIP2 were associated with an increase in Synj1 [491], a PIP2-degrading enzyme [507] significantly upregulated in AD brains [508] and associated with reduced internalization and degradation of Aβ [509]. Synj1 appeared upregulated in the neocortex and hippocampus of *APOE4/4* mice. These mice have significant memory impairment, abrogated by the genetic knockdown of Synj1 [491]. Cao and colleagues identified the miRNA miR-195 as a candidate in regulating the apoE-synj1-PIP2 pathway. The overexpression of miR-195 restored cognitive deficits and ameliorated amyloid and p-tau pathology in *APOE4* individuals [510]. This study offers a new potential therapeutic approach to the regulation of apoE4 and its effect on autophagy in AD.

Altogether, different studies established a clear link between autophagy and *APOE4*, which may be one of the main cellular processes underlying the detrimental effect of *APOE4* in AD. Although the mechanisms involved are not fully understood, different shreds of evidence show the alteration of relevant elements such as mTOR1 or PIP2. Dissecting the components involved in this complex process may offer new and hopeful therapeutic approaches in AD treatment. A summary of the effects of the APOE4 genotype on the AD-related changes can be seen in Table 5.

**Table 5:** Effects of the APOE4 genotype on AD pathological features

	**ApoE4-induced changes related to AD**	**References**
**Amyloid-β**	Increased plaque load and density	-Humans: [160, 339, 340] -Mice: APP^V717F^ x humanized APOE4 [66, 341], PDAPP/TRE4 mice [511], E4FAD mice [512]
	Increased CAA formation	-Humans: [513, 514] -Mice: Tg2576 x hAPOE4 KI [344]
	Increased Pittsburgh compound B positive amyloid burden	-Humans: [346, 347]
	Reduced Aβ_42_ levels in CSF	-Humans: [346, 348]
	Enhanced Aβ fibril formation in vitro	-Aggregation studies [291] -In vitro studies: [351, 352]
	Increased intracellular Aβ generation/Aβ secretion	-Cellular models: [358, 359]
	Reduced neprilysin and IDE expression	-Humans: [365, 366]
	Reduced cellular clearance of Aβ	-In vitro: [364, 367, 368]
	Impaired Aβ removal via BBB	-Humans: [391] -mice: APP/PS1 mice [435] -Human and APP knock-in mice: [374]
	More severe tau pathology	-Humans: [390]
**Tau**	Increased tau-induced neurodegeneration	-Mice: P301S/APOE4 knock-in mice [397, 401]
	Enhanced tau phosphorylation	-In vitro: [399, 402, 515] -Mice: E4FAD [516]
	Increased cleavage of C-terminal truncated ApoE fragments (linked to increased p-tau and tau tangles-like structures)	-In vitro: [383, 517]
**Synapses**	Reduced neuronal outgrowth and synaptic density	-In vitro: [447–449] -Mice: APOE4 knock-in [234]
	Impaired synaptic plasticity (Long term potentiation)	-In vitro: [452] -Mice: APOE knock-in [453]
	Poor learning and memory	-Mice: APOE4 knock-in [456–458]
**Autophagy/Endosomes**	Decreased/impaired autophagy and mitophagy	-In vitro: [470] -Mice: APOE4 knock-in [486]
	Dysregulated endosomal-lysosomal pathway	-Mice: APOE4 knock-in [518]
	Enlarged endosomes	-Humans: [481] -Mice: APOE4 knock-in [518]
	Reduced mRNA levels of autophagy markers	-Humans: [489]

### Connecting *APOE* with other non-genetic AD-associated risk factors

#### Age

Aging is considered one of the greatest risk factors implicated in developing neurodegenerative diseases, including AD [519, 520]. Normal cognitive aging is related to neurocognitive changes, such as alterations in processing speed, loss of attention, memory-related deficits, decline in verbal fluency, visuospatial abilities, and executive functioning, whereas some of the structural and functional changes include gray and white matter volume decline [521, 522]. Nonetheless, the exact mechanisms and molecular pathways connecting aging and the development of neurodegenerative diseases are yet unknown. In addition, it is still not determined the connection between the *APOE* genotype, aging, and the development of neurodegeneration. An age-dependent increase of apoE CNS levels has been documented, and these levels can be affected by the amyloid pathological status [201]. A case–control study comparing the age-specific association between the *APOE* genotype and AD in the CSF of more than a thousand AD cases reported that the highest impact of *APOE4* on AD risk was between the ages of 65 and 70 [523].

Brain aging is related to immunosenescence and low-grade inflammation (inflammaging), to which activated macrophages and monocytes will contribute and promote neuroinflammation, a key event in chronic age-related neurodegenerative diseases [524, 525]. Similarly, systemic inflammation is also a predictor of brain aging [526, 527]. Previous studies have reported how the hypothalamic-derived release of inflammatory mediators controls systemic aging [528]. Quantifying the inflammatory-protein concentration in the CSF and human plasma of AD patients at different AD stages showed a correlation of CSF inflammatory changes with Aβ and tau levels, neurodegeneration, and cognition [529]. The *APOE4* genotype is associated with lower C-reactive protein levels, which in turn, correlates with greater *APOE*-related AD risk [526]. Conversely, the expression of the *APOE2* allele has been connected with increased life longevity and protecting against age-induced cognitive decline independently of AD pathology [530].

The study from Zhao and colleagues evaluated in *APOE* targeted-replacement mice the effect of age, sex, and the *APOE* genotype in the brain transcriptome and serum metabolome, in which they found a strong impact of age in the brain transcriptome together with brain-specific elevated SerpinA3n levels in *APOE4* mice [531]. Serpina3n is a serine protease inhibitor, it is the mouse orthologue of the human alpha-1antichymotrypsin, and an inflammatory protein overexpressed in the AD brain, able to induce tau hyperphosphorylation in neurons [532, 533]. SerpinA3n is also involved in traumatic brain injury (TBI) and multiple sclerosis (MS); during TBI, SerpinA3n deficiency aggravates neuronal apoptosis and cognitive disfunction after hippocampal stab injury, and progressive MS patients present increased SerpinA3n protein levels have [534, 535].

Aging is also associated with chronic glial activation, which correlates with age-related cognitive decline and neurodegeneration [536]. Thus, microglial depletion and repopulation has been proposed as potential therapy to normalize chronic immune activation [537–539]. Another example would be the study from Coleman and colleagues in which microglial depletion and repopulation in primary organotypic hippocampal slice cultures normalized persistent pro-inflammatory gene expression [540]. Interestingly, it has been noted that the brain can only perform one whole repopulation event at a time, however, with sufficient time, it can ultimately repopulate after repeated depletion events [541].

#### Sex

Female sex, together with advanced age and the expression of *APOE4* genotype, constitutes one of the most relevant risk factors for the development of AD. In the United States, two-thirds of the diagnosed AD cases correspond to women [542, 543]. This fact is often associated with the higher longevity of women [544]. On the other hand, recent translational imaging studies documented an AD-phenotype characterized by low metabolic activity and high Aβ deposition in perimenopausal and postmenopausal women aged 40–60 years old [545]. This prior study also evidenced an increased Aβ deposition in women carrying the *APOE4* genotype. Likewise, it was recently confirmed the sex-specificity between AD biomarkers and CSF apoE, where baseline CSF apoE levels in women were significantly associated with Aβ and tau levels [417], which does not entirely align with results from previous studies [546, 547].

Neuroinflammation is a strong contributor in AD pathophysiology; however, the impact of glial cell sexual dimorphism in the neuroinflammatory response is still yet fairly unknown. Studies characterizing the neuroinflammatory response in male and female C57Bl/6 N mice suggest that type 1 interferons could be one of the potential sex-mediating cytokines that influencing cognition [548].

Microglial sexual dimorphism has been taken under consideration in the context of AD and synaptic function, ischemic stroke, and traumatic brain injury [549–552]. Of note, microglial responses to environmental challenges occur in a sex- and time-dependent manner. A mouse and human study addressing the microbiome’s influence in prenatal and adult microglia revealed that antibiotic treatment of adult mice led to sexually biased microglial responses [553]. Further on, regarding astrocytic implications in neuroinflammation, sex-mediated differences in the astrocytic response towards an inflammatory stimulus have been described in primary astrocytic cell lines [554, 555]. ApoE can target synapses and impact neuronal activity differently if released by neurons or astrocytes [460]. Live-calcium imaging techniques in vitro cultured neuron models have evidenced that neuron-released apoE induces the highest neuronal firing in the presence of apoE3 and not apoE4, whereas astrocytic-derived apoE induces the strongest neuronal firing with apoE4 [460]. Additionally, it has been reported in *APOE4* astrocytes from male but not female *APOE*-targeted replacement mice higher calcium hyperactivity [556].

The impact of sexual differences in glial physiology have been reviewed by Crespo-Castrillo and colleagues [557]. Further studies taking into account the cellular sexual dimorphism will be of great value to further unravel the interaction between sex and the *APOE* genotype in neurodegenerative diseases, particularly in AD.

#### Diet

Dietary habits and lifestyle strongly impact gut microbiota composition, and the gut microbiome is gaining increasing relevance as a modifier of the susceptibility and progression of neurological and neurodegenerative diseases [558–560]. Evidence between healthy dietary patterns, their potential anti-neuroinflammatory action, and their influence on age-related cognition has been reviewed by McGrattan and colleagues [561].

Multiple studies have shown that the gut microglioma and the gut-brain axis play a significant role in brain function and impacts neuroinflammation and AD progression [562, 563]. Additionally, some epidemiological studies propose a link between type 2 diabetes mellitus (T2DM) and AD [564, 565].

AD and T2DM share as a common feature the presence of misfolded proteins, Aβ and islet amyloid polypeptide, respectively [566]. T2DM is one of the prime risk factors for AD development, together with the expression of the *APOE4* allele. Nonetheless, the precise mechanisms by which the *APOE* genotype and diabetes contribute to AD risk are still undetermined. EOAD and LOAD are characterized by different metabolic connectivity features [567]. It has been proposed that Insulin influences AD pathology by directly interacting with Aβ peptides [568, 569].

Insulin resistance and impaired glucose metabolism are two extensively reviewed AD features [568]. The association between the *APOE* genotype polymorphisms and the risk of suffering T2DM and cardiovascular disease have also been reported, pointing at *APOE4* as a risk factor for T2DM and cardiovascular diseases [565, 570–572]. *APOE* influences insulin resistance and glucose metabolism in the brain [336, 573, 574]. TaqMan arrays studies in *APOE* transgenic mice revealed that brains from *APOE4* mice are the most deficient profile on glucose uptake and metabolism [575]. *APOE* isoforms cause differential effects on the functioning of the brain insulin receptor. It has been observed that apoE3 binds with higher affinity to the insulin receptor than apoE4 [576], and a single insulin injection in *APOE4*-carrying mice induced increased activation of the insulin upstream brain signaling pathways, such as the Akt pathway, coupled to higher phosphorylation levels of the Ser202 tau residue [577]. It has also been reported in primary neuron studies that apoE4 interacts with the insulin receptor, trapping it in the late endosomes and impairing its trafficking, thus causing impaired insulin signaling, insulin-coupled mitochondrial respiration and glycolysis [578].

A high-fat diet has been shown to increase gliosis in *APOE3* mice but not in *APOE4* mice [579]. Precision nutrition interventional approaches have been suggested for *APOE4* carriers [580]. A systematic review of the clinical records from the United States National Alzheimer Coordinating center revealed that diabetes is linked to earlier cognitive decline during aging in *APOE2* and *APOE3* carriers, but not in *APOE4* carriers, in which the effect of diabetes was negligible, perhaps because *APOE4* carriers are already more prone to suffer vascular impairment and cognitive decline [581]. The prior study also observed lower AD neuropathological hallmarks in *APOE3* carriers [581]. Thus, future studies evaluating the impact of diet and the *APOE* genotype in AD pathology will be of great value.

#### Physical exercise

Several robust studies have shown the health benefits of physical activity in cardiopulmonary function, neuroplasticity, cognition, and bone density [582, 583]. Interestingly, related to bone density, it was recently reported the discovery of a specialized bone-cell progenitor that supports immune cell generation in response to physical exercise [584]. Physical activity has also been associated with a lower risk of developing dementia [585–589]. However, these studies are inconclusive to what extent exercise may prevent or postpone AD development [590–593]. Anthropologists hypothesize that the evolution of increased physical activity 2 million years ago might have been a prerequisite for human cognitive longevity due to the reduction in amyloid pathology and vascular burden of *APOE4* [53]. In that context, it is intriguing whether or not the *APOE* genotype impacts the association between a physically active lifestyle and the development of dementia, particularly in AD.

A wide array of studies evaluated the impact of physical exercise intervention upon multiple aspects of AD pathology. Various meta-analyses have studied the effect of exercise in cognitive function, suggesting that exercise training could delay cognitive function decline in AD-risk and AD patients [594, 595]. Other authors have addressed the impact of physical exercise upon various AD biomarkers, such as hippocampal volume, in which some studies observed volumetric increases in the hippocampus, whereas others failed to do so [596, 597]. Alternative AD biomarkers evaluated regarding the impact of physical exercise are Aβ turnover, cerebral blood flow, neurotrophin release, and inflammation [598–600].

Although vascular dementia and AD are considered independent disorders, vascular dysfunction also plays a key role in AD pathogenesis [601–603]. Indeed, early exercise intervention in a model of chronic cerebral hypoperfusion led to normalized capillary density and decreased leukocyte rolling in chronic hypoperfused mouse brains, paired with a decrease in hippocampal microglial activation and an improvement of microvessel-covering by astrocytes [604].

Few exercise studies discriminate between *APOE* genotypes, and among those who do, results are diverse, as reported by recent systematic reviews [605, 606]. Regarding the risk of getting diagnosed with AD, some studies show that the lower risk of developing all-cause dementia and AD among highly active persons is more pronounced in *APOE4* non-carriers [586, 587, 607, 608]. In contrast, other studies demonstrate that this association is more valid for people carrying the *APOE4* [593], whereas results reported by our research group, and others, do not reveal any impact of the *APOE* genotype on this relationship [590, 609, 610]. Regarding cognition, some studies find that the association between physical activity and preserved cognition is more noticeable among *APOE4* carriers [610–612]. Opposingly, others reveal an attenuation or even absence of this relationship in *APOE4*-carriers [608, 613, 614], while some display no impact of the genotype [609, 615–617].

Further, neuroimaging studies indicate that the benefits of physical activity on cortical activation, prefrontal cortex connectivity, and preservation of the hippocampal volume might be limited to *APOE4* carriers [618, 619]. Moreover, even among APOE4 carriers, a randomized controlled exercise intervention only improved hippocampal blood flow in those who initially suffered from hypertension [620]. Considering the amyloid pathology, Head and colleagues demonstrated that a more sedentary lifestyle was associated with higher amyloid PET, but only in *APOE4*-carriers [621]. Their findings align with a recent systematic review, suggesting that the association between physical activity and the amyloid burden is more evident in *APOE4* carriers [605]. Still, exercise intervention in patients with mild AD did not affect tau or amyloid biomarkers in CSF, regardless of the apoE status [622], and this lack of impact correlates with another study [623].

The involvement of neuroinflammation in the pathophysiology of AD has caught increasing interest during the past decade. Exercise affects the inflammatory status peripherally, and several studies suggest that exercise may also reduce neuroinflammation [600, 624, 625]. Still, the impact of the *APOE* genotype in neuroinflammation is not yet fully elucidated. Jensen and colleagues detected a stabilizing effect on plasma IFNγ following exercise only among patients with mild AD who were *APOE4* carriers [624]. Experimentally, *APOE* deficiency compromises the microglial response to amyloid plaques [236], and exercise may prevent the age-related decrease in astrocytic *APOE* expression, which has been liked to microglial activation, an effect absent in *APOE* knockout mice [626]. Therefore, there is still a knowledge gap regarding the impact of *APOE* on the neuroinflammatory effect of exercise. Increased insight in this area could potentially open up new treatment strategies combining exercise interventions with future pharmacological therapies to target specific neuroinflammatory pathological events early in AD patients depending on *APOE* genotype. A limitation is that nearly all of the investigations made in this field only consider the influence of *APOE* on dementia risk and do not examine if this genotype affects exercise behavior. A study from the UK biobank that included over 350 000 individuals revealed that the *APOE4* variant was the most strongly linked gene to moderate and vigorous physical activity [627]. Likewise, a study comparing over 200 athletes with controls found an implication of the *APOE* single nucleotide polymorphism (SNP) rs429358 in the modulation of the distinction between power athletes and sedentary individuals [628]. On the other hand, in our previous study that included 10 000 individuals, the *APOE4* genotype was equally distributed between groups with different levels of lifestyle physical activity [590]. Further, if participants in a study are aware of their *APOE4* genotype and what that implies in terms of long-term risks, they may be more motivated to become more physically active, which may influence their outcomes.

The discrepancy between studies may be attributable to several factors, such as the type of exercise assessment and outcome measurement. For instance, a recent systematic review concluded that most studies with conflicting results used outcomes such as clinical diagnosis, which are not sensitive to preclinical cognitive fluctuations [606]. Timing of exercise intervention or physical activity lifestyle assessment are also relevant parameters to consider since physical activity may need to occur when it is still possible to prevent the pathological process. This prior fact may be significantly important for individuals with genetic predispositions, such as the *APOE4* carriers. The study by Krell-Roesch and colleagues is a representative example of when physical activity in midlife seems to preserve memory function, particularly in *APOE4*-carriers, whereas only non-carriers benefitted from engaging in physical activity later in life [629]. In addition, it is necessary to note that studies with shorter follow-up times (e.g. > 5 years) risk finding associations due to reverse causation since individuals in a prodromal disease stage may engage less in physical activity [586, 590, 630, 631]. Conversely, the association between physical activity and a lower risk of mortality in several diseases ultimately leads to an extended lifespan where neurodegeneration eventually will develop. Therefore, it is crucial to discuss the time point when physical exercise significantly decreases the risks and improves the patient’s life quality. Being physically active may prevent dementia development only during a specific time for individuals with genetic constraints such as *APOE4*, and effects may be lost with very long follow-up but still be clinically relevant.

## Current AD therapies and clinical trials. Targeting *APOE4*

Over the last few years, many experimental studies have focused on animal models targeting *APOE4*, showing promising results. These include the increase of apoE levels using bexarotene, apoE mimetics, blocking the apoE-Aβ interaction, *APOE* silencing, and the use of genome editing to switch from *APOE4* to *APOE2* or *APOE3* isoforms. These advances have been carefully reviewed in Lancet Neurology in 2021 (see review [632]). Nonetheless, despite promising results in animal models, there is a low number of completed or ongoing clinical trials [632].

Two clinical trials have focused on increasing apoE levels by administering bexarotene, an RXR agonist. Oral administration of bexarotene in APP/PS1 mice enhanced Aβ clearance, reduced Aβ plaques area, and improved cognitive deficits [633]. In humans, bexarotene showed poor CNS penetration in healthy subjects and resulted in a modest increase of apoE levels in CSF [634]. In phase 2 clinical trials, bexarotene administration showed a significant reduction in brain amyloid in *APOE4* noncarriers, with no changes in *APOE4* carriers, so the primary outcome of the clinical trial was negative [635]. Another clinical trial followed a similar approach with probucol (NCT02707458), a non-statin lipid-lowering drug; however, there are still no results published to date [632].

In addition to those mentioned above, two Phase 1 ongoing clinical trials show novel *APOE4* therapeutic approaches in patients with AD or MCI (NCT03887741 and NCT03634007). The first trial focuses on the genetic switch of *APOE4* to *APOE2* through adenoviral vectors (AAV). The trial will assess the safety of the intracisternal administration of the AAV in patients *APOE4* homozygotes with AD (NCT03634007). This approach showed promising results in an *APOE4* knock-in model, increasing apoE lipidation and decreasing endogenous Aβ [636, 637]. The second clinical trial aims to assess the safety of plasmapheresis from young *APOE3* homozygotes into *APOE4* homozygotes with MCI (NCT03887741). Both clinical trials are still in the recruitment phase.

The use of immunotherapy (e.g., aducanumab, gantenerumab, lecanemab) is one of the most promising recent advances in treating AD. However, their implementation is not without controversy. The administration of these antibodies potentially induced amyloid-related imaging abnormalities (ARIA), associated with the development of brain hemorrhages. The cause of this adverse response is still unknown. Nevertheless, different works showed an exacerbated ARIA response in *APOE4 carriers* [638, 639]. Immunotherapy against AD opens another attractive therapeutic alternative, the use of anti-apoE antibodies. To date, studies in animal models have shown that the use of anti-apoE monoclonal antibodies such as HJ6-3 and HAE-4 reduces Aβ and apoE levels in the brain, along with an improvement in cognitive performance [640–643]. Specifically, the use of the HAE-4 antibody that identifies low-lipidated apoE reduced both parenchymal Aβ plaques and Aβ in the cerebral vasculature without vascular complications associated with Aβ antibodies [643]. These studies evidence the potential interest of this strategy, which could be interesting to test in clinical trials in the coming years.

The knowledge about *APOE’s* role in AD has increased significantly in recent years. Different animal model studies highlighted the potential therapeutic implications of *APOE*, but many aspects require further investigation. The advent of new therapeutic approaches such as gene editing or immunotherapy, together with all the knowledge accumulated to date, implies that this is only the beginning of a promising research field.

## Implications of *APOE* in other neurodegenerative diseases

The main hallmark of neurodegeneration will be the loss of neuronal structure and connectivity that can lead to altered movement (ataxia) or dementia [644]. Among the primary mechanisms involved in most neurodegenerative diseases are abnormal protein accumulation, immune system activation, and neuronal death [645]. These three mechanisms are tightly connected, and their interaction determines most of the detrimental events involved in the development of the pathologies.

There is growing evidence that prolonged brain neuroinflammation and cytokine release boosts neurodegeneration and exacerbates Aβ and NFT pathology and the progression of other neurodegenerative diseases [221, 646, 647]. Additional events contributing to neurodegeneration include oxidative stress events, mitochondrial dysfunction, Golgi apparatus fragmentation, disruption of axonal transport, neurotrophins dysfunction, and cell death mechanisms such as apoptosis and necrosis [648, 649].

Despite the vast array of neurodegenerative diseases described in humans, there some similarities among them. Perhaps the most outstanding are the abnormal protein accumulation, neuronal death, and the activation of the innate immune system. Innate immune system activation in the brain is commonly driven by glial cells, especially microglia. The main focus of this review is the role of *APOE*, and its isoforms, during neuroinflammation observed in neurodegenerative diseases, with particular attention to its role in neuroinflammation. A tremendous scientific effort in the neurodegenerative diseases field is centered on resolving the puzzle of AD pathology. However, other diseases characterized by overt neuroinflammation can also shed light on the impact of the *APOE* genotype and how the brain works in disease conditions, and due to the similarities before mentioned, apply that knowledge to other research areas.

In this section, we are exploring the importance of the *APOE* genotype in neurodegenerative diseases other than AD. A common feature of the brain diseases in this section is the abnormal protein accumulation within the neurons or an acute detrimental event. The intraneuronal protein accumulation could lead to a different role of apoE compared to the mechanism involving apoE and the immune system response. For instance, the role of *APOE* in the neuronal lysosome or autophagy regulation might play a more significant role than the recently described role of *APOE* in microglial phenotype activity [269, 650, 651]. However, stroke or traumatic brain injury elicits a robust inflammatory response mainly driven by microglial cells. Thus, understanding the dual role of apoE in the regulation of the immune system or the intraneuronal metabolism is a great challenge for the scientific community nowadays.

**Fig. 3:**
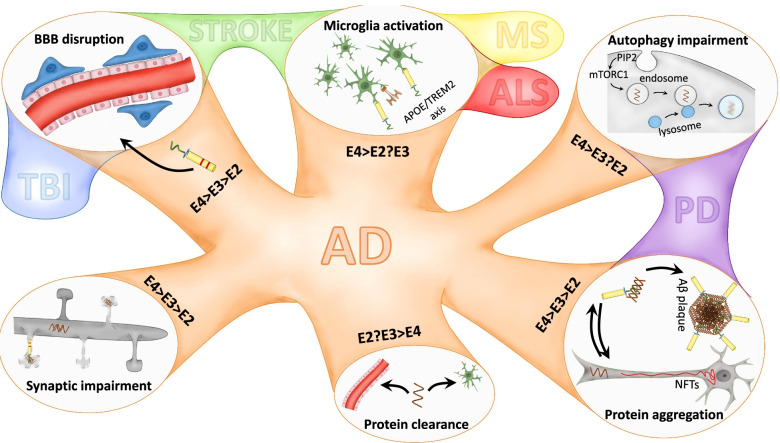
Mechanisms involved in apoE-related neurodegeneration. The influence of the *APOE* genotype has been associated with CNS diseases such as Alzheimer’s Disease (AD), Parkinson’s Disease (PD), Traumatic Brain Injury (TBI), Stroke, Amyotrophic Lateral Sclerosis (ALS), and Multiple Sclerosis (MS). In particular, *APOE4* promotes synaptic impairment, amyloid-β (Aβ) protein seeding, aggregation, Aβ-plaque formation and defective protein clearance in AD. *APOE4* will contribute to additional pathological features characteristic of AD, but also present in other CNS pathologies such as, for instance, impairment of the autophagy/endosomal system, altered microglial reactivity and neuroinflammation, and blood–brain barrier disruption

Altogether, *APOE* might share common mechanisms of action among neurodegenerative diseases with an abnormal protein accumulation or an acute detrimental event. In the coming section, we will explore in more detail the latest finding on the role of *APOE* in Parkinson’s disease and other synucleopathies, stroke, TBI, ALS, or MS (Fig. 3).

### Parkinson’s disease

Parkinson's Disease (PD) constitutes the second most prevalent neurodegenerative disease. PD characterizes by the intracellular accumulation of proteinaceous aggregates called Lewy Bodies (LBs). LBs constitute rounded intracellular aggregates mainly composed of α-syn, yet, additional proteins and lipids can also be present, conferring these aggregates a highly complex composition, for further details regarding structural, morphological, and biochemical properties of LBs, refer to the review from Fares and colleagues [652]. α-syn is a 140 amino acid protein composed of a C-terminal acidic domain, a central domain highly hydrophobic and involved in aggregation, and an N-terminal domain with lipid-binding similar to apoE’s lipid-binding domain [653]. Remarkably, all six known mutations in the SCNA gene that leads to familial PD are located in the N-terminal domain [654]. This domain possesses hexameric repeats of 11 amino acids, characteristic of apolipoproteins, which confers α-syn lipid-binding abilities [655]. The apoE-cholesterol pathway differentially affects both AD and Lewy Body dementia (LBD), and the relevance of cholesterol in Lewy Body pathology has been recently reviewed [656–658]. Firstly, α-syn is known to change its morphology in response to lipid binding; secondly, most of the known PD-related mutations influence cholesterol levels; and lastly, α-syn deficiency leads to increase neuronal lipids in mice [659]. Interaction between α-syn and apoE protein has also been documented. In vitro experiments showed that all isoforms of apoE, but apoE4 in particular, increases α-syn aggregation [660]. In human samples, fragments from apoE protein have been recently discovered to form part of the majority of the LBs [661]. Additionally, CSF lipid vesicles from PD patients, but not control patients, contain both apoE and α-syn, indicating that increased neuronal cholesterol efflux could be a late event in PD [662].

In PD, protein aggregates are primarily found in the dopaminergic neurons of the Substantia Nigra; other areas such as the cortical region, hippocampus, amygdala, olfactory bulb, or gut nervous system, can be affected in diverse subtypes or stages of the disease [663]. Importantly, LBD pathology is not only present in PD, but other related diseases like PD with dementia (PDD), from which is practically indistinguishable when its fully clinically developed, and LBD, that commonly has some degree of concomitant AD [664, 665].

From a genetic perspective, *APOE* mutations have long been related to a higher risk of developing PD. However, several studies showed discrepancies suggesting that *APOE* mutations may be more related to a higher risk of worsening cognitive function and not to motor degeneration [666–671]. Nonetheless, it has been detected the presence of apoE-positive neuromelanin-containing dopaminergic neurons in substantia nigra of PD patients [662].

Neuroinflammation has long been considered a central player in PD onset and development [672]. However, despite the recent description of microglial phenotypes associated with several neurodegenerative diseases, no microglial phenotype has been associated with PD [673]. Moreover, albeit the *APOE* relevance for the neurodegenerative phenotypes of microglia, data on the effect of *APOE* variants in PD-associated microglia are scarce. Recently, Choi and colleagues described a microglial phenotype associated with microglial α-syn clearance [674]. This phenotype has several transcriptomic similarities with other neurodegenerative phenotypes previously described by Krasemann and colleagues [673], such as upregulation of *Axl*, *Lpl*, and importantly, *apoE*. Thus, investigating the role of apoE isoforms in this microglial phenotype could help better understand the role of apoE and microglia in PD onset, dementia development, and α-syn clearance.

Synucleinopathies and amyloidopathies are commonly found simultaneously in the brain of PD and AD patients, hindering the effect of *APOE* mutations in synucleinopathies' progression. Lately, *APOE* genetic mutations have been considered a risk factor for developing dementia in all synucleinopathies subtypes, including PD, but not related to PD onset [675]. The study by Dickson and colleagues, including more than 600 hundred PDD and LBD post-mortem samples, showed that severity of LB pathology was associated with *APOE4* variant independently of the severity of concomitant AD, yet, the analysis only included cortical regions [676]. Two additional studies recently showed implications of apoE4 in synucleinopathies not related to AD [677, 678]. Goldberg and colleagues propose that *APOE4* could be related not only with the severity of LB pathology but also with the extension of the pathology to other areas, in comparison with *APOE2* or *APOE3* variants where the pathology is more prone to be restricted to the midbrain in mouse models [679]. Prior results could suggest that *APOE4* is related to the progression of LB pathology from the classical PD to related LB diseases like PDD or DLB. Overall, even if *APOE4* is not considered as a risk factor for developing PD, recent studies evidence that it is risk factor for developing dementia, worsens cognitive function, and can be related to the spreading of LB burden from the midbrain to other areas, perhaps due to distinct roles of *APOE* isoforms in microglia-associated α-syn clearance.

### Other synucleinopathies

In addition to its association with an increased risk of AD, we have previously addressed in this review that *APOE4* is also an outstanding genetic risk factor for LBD [680]. Analogously to AD, the *APOE2* variant appears protective and delays LBD onset [680]. Furthermore, the presence of apoE4 negatively affects cognitive performance in PD patients and increases the risk of dementia in pure synucleinopathies (with limited AD co-pathology) [666, 681]. There are no studies associating *APOE4* with altered risk of suffering oligodendrocytic synucleinopathy multiple system atrophy (MSA), although astrocyte-secreted apoE4 unexpectedly was found to reduce the uptake of α-synuclein in cultures of oligodendrocytic cells [682].

The implications of *APOE4* in synucleinopathies are still poorly understood, but recent studies suggest that the *APOE* gene regulates α-syn brain pathology independently of its effects on AD-related Aβ and tau pathology and that specifically *APOE4* exacerbates α-syn pathology [683, 684]. A study led by Paslawski reported that α-synuclein could co-localize with apoE-containing lipoparticles in the CSF, and that apoE CSF levels were higher in patients with early PD compared to controls [662]. Intriguingly, *APOE4* has also been associated with higher levels of CSF α-syn in patients in the AD continuum where patients with mild cognitive impairment (MCI) who, after 24 months follow-up fulfilled the clinical criteria for an AD diagnosis, exhibited higher CSF α-syn levels in an *APOE4* dose-dependent manner [685]. The same study further reported that higher CSF α-synuclein levels were associated with brain Aβ burden as assessed by PiB-PET in carriers of autosomal-dominant AD mutations that in addition carried the *APOE4* gene variant. Indeed, accumulating studies propose α-synuclein to be an active player in not only synucleinopathies but also in AD pathophysiology in which *APOE4*/apoE4—α-syn interactions may play a still-to-be defined role [686].

### Traumatic Brain injury

According to the United States CDC, TBI can be caused by a bump, blow, or jolt inflicted to the head or by a penetrating head injury that will cause a disruption of the normal brain function and will have short- and long-term effects on health [687, 688]. TBI is considered as one of the risk factors for the development of AD and it is associated with an earlier dementia onset [689–691]. The recovery rates among people that have suffered TBI differ among individuals, and multiple metanalyses point out a relationship between the *APOE4* genotype and increased risk of poor long-term outcomes following TBI [692, 693].

The relationship between *APOE* and TBI has been extensively studied. RNA-sequencing studies together with bioinformatic analysis have associated TBI with neuroinflammation and neurodegeneration, outlining *apoE*, *Mapt*, and *Trem2* as differentially expressed genes and astrocytes and microglia as key cells involved [694–696]. The study from Giarratana and colleagues documented that PKCε activation and consequent increase in BDNF levels decreased inflammation and microglial activation in *APOE4* knock in mice subjected to mild TBI [697].

BBB dysfunction constitutes one of the pathophysiological events present in TBI and has been reported in TBI patients [698]. ApoE is implicated in preserving tight-junctions integrity at the BBB in an isoform-dependent manner, in which studies with *APOE4*-KI mice have reported higher BBB permeability than in *APOE3*-KI mice [377, 699]. It has been documented that *APOE3* and *APOE4* mice react similarly to TBI, albeit *APOE3* mice present higher BBB recovery rates than *APOE4* mice [700]. Moreover, both *APOE4* homozygous and heterozygous individuals are characterized with BBB breakdown in brain areas such as the hippocampus and the medial temporal lobe that may contribute to cognitive decline, and apoE4 accelerates BBB breakdown [375, 701, 702].

Early studies on TBI reported an increase of tau levels in the CSF of TBI patients. Parallelly, an increase of intraneuronal Aβ_1-42_ accumulation in the terminal ends of dystrophic axons has been observed after TBI. Nonetheless, the specific role of intraneuronal Aβ in TBI is still unknown. A study from Abu Hamdeh S. and colleagues reported increased levels of Amyloid oligomers/protofibrils and Aβ_1-42_ in the brain tissue of postmortem TBI patients heterozygous for the *APOE4* allele and concluded that soluble intermediary Aβ tends to aggregate more rapidly after TBI in the presence of apoE4 [703, 704]. Interestingly, in the study conducted by Montagne and colleagues, authors did not observe a correlation between the BBB breakdown increase in *APOE4* carriers and the Aβ or tau CSF levels, yet, prior studies have reported increased phosphorylated tau levels in *APOE4* mice after TBI in the CSF and hippocampal region [705, 706].

Repeated TBI is known to initiate cerebrovascular diseases, and can lead to chronic traumatic encephalopathy (CTE). Historically CTE is known as dementia pugilistica, it was first described in boxers in 1928, and closely related to cerebrovascular dysfunction [707]. Neuropathologically, CTE is characterized by NFTs similar to those found in AD and diffuse Aβ plaques, particularly in *APOE4* carriers [708–710]. A postmortem study of 1275 human brains evaluated the link between the *APOE* genotype and the development of cerebrovascular disease, concluding that the *APOE2* genotype was not protective and significantly increased the risk of suffering hemorrhages when cerebrovascular amyloid angiopathy was present [711]. Therefore, a better understanding of the mechanisms involving TBI and *APOE* could be of great interest to discover possible disease-modifying biomarkers to halt AD progression.

### Stroke

Stroke consists of a disruption in the brain’s blood flow either due to a lack of blood flow (ischemia) or to bleeding (hemorrhage) [712]. The global burden of ischemic strokes is fourfold higher than hemorrhagic [713].

The *APOE* genotype influences the age onset of stroke and its severity. Notably, the expression of the *APOE4* allele is related to delayed recovery of verbal memory function and a reduced EC volume evaluated one year after the ischemic stroke event [714]. *APOE4* has been shown to accelerate the risk of developing stroke-associated dementia, possibly related to the implication of *APOE4* in BBB dysfunction, as commented in the prior TBI section of this review [715, 716]. Similarly, a meta-analysis carried out by the Swedish Heart and Lung foundation with combined information including the *APOE* alleles, sex, age of onset of ischemic stroke, stroke severity, and outcome, established a link between the *APOE4* allele expression and younger age of stroke onset, so as a link between *APOE4* and favorable stoke outcomes, whereas *APOE2* allele male carriers were directly related with poor stroke outcomes. Nonetheless, the authors did not observe any association between the *APOE* alleles and stroke severity [717]. In a Chinese population meta-analysis, the *APOE4* genotype could also predict different types of strokes, including ischemic stroke, intracerebral hemorrhage, and subarachnoid hemorrhage [718].

The neuroinflammation-related effects of hypoxic-ischemia in the brain have previously been described, leading to a microglial cell proliferation increase, astrocyte reactivity, and release of pro-inflammatory cytokines [719, 720]. Additionally, a unique subtype of microglial cells in the thalamus of C57BL6J mice subjected to ischemic stroke was recently reported. This microglial subtype is transcriptomically characterized by upregulated *apoE*, *Axl*, *LpL*, *CSF1*, and *Cst7* levels [721]. Related to the neuroinflammatory response during stroke, Tomas Deierborg’s group demonstrated in an in vivo stroke model the deleterious role of gal-3 dependent inflammatory response, in which the lack of gal-3 reduced the infarct area in the stroke-induced model, and intranigral injection of LPS in gal-3 knock-out mice reduced neuronal death compared to gal-3 WT mice [722].

Endothelial cells and astrocytes will also play a significant role after stroke; Jun Xiang and colleagues evaluated how astrocyte-endothelial cell interaction could influence stoke pathogenesis in an oxygen and glucose deprivation-reoxygenation (OGD-R) model and reported that astrocyte-derived apoE activated the PI3k/eNOS pathway in endothelial cells as protection mechanism [723].

Ischemic stroke can induce white and grey matter damage, limits axonal regeneration and remyelination, and might lead to vascular dementia and Alzheimer’s disease [724, 725]. The *APOE2* genotype has also been associated with white matter disease in ischemic stroke patients, together with *APOE4* [726, 727]. Related to this, Hayden Y. and colleagues aimed to better characterize the interaction between white matter ischemic lesions, the *APOE4* genotype, and amyloid pathology in AD using E4FAD mice subjected to a subcortical ischemic stroke. The authors did not observe any effect of the 5xFAD transgene on the stroke lesion, nor in the cortical amyloid deposition between the sham and stroke E4FAD mice, despite that, there was a high microglial reactivity and cortical neuron damage in the stroke-induced E4FAD mice, suggesting that the microglial reactivity induced by the subcortical ischemic lesion may be promoting Aβ clearance. Still, this study was limited to early time points (7 to 14 days post-stroke assessment) and young mice [728].

### Amyotrophic lateral sclerosis

Amyotrophic lateral sclerosis (ALS) is a progressive and fatal neurodegenerative disease characterized by deposition of aggregated proteins that will affect motor neurons integrity. Motor-neuron dysfunction will lead to muscle weakness until complete loss of voluntary muscle movement, including breathing, speaking, eating difficulties, and some individuals are also diagnosed with frontotemporal dementia [729, 730]. Although ALS etiopathology is multifactorial, the most prevalent mutations present in ALS are in the proteins Superoxide-dismutase1 (SOD-1) and TAR DNA-binding protein 1 (TDP-43) [731, 732]. Defective energetic metabolism, disruption of iron homeostasis, and neuroinflammation are also components of ALS pathogenesis [733]. ALS-related neuroinflammation comprises reactive microglia and astrocytic cells, together with T-cell and macrophage infiltrations in the CNS, and activation of inflammatory pathways [734–736]. As mentioned in previous sections of our manuscript, the microglial *APOE*-dependent molecular signature identified by Krasemann and colleagues observed as well in ALS models [269].

Additionally, many therapeutic approaches have targeted astrocytes, the main apoE-producing cells, to potentially stop ALS progression [737]. In the early two-thousands, the *APOE* genotype gained attention as a potential marker for ALS onset and progression, leading to conflicting results. Some studies suggest the *APOE2* genotype to be protective, and *APOE4* detrimental for ALS onset, whereas others suggest that the *APOE* genotype does not influence ALS clinical course [738, 739]. More recent studies evaluating the influence of the *APOE* genotype in brain metabolism during ALS showed that the *APOE2* genotype correlates with hypometabolism in the brain motor areas usually affected by frontotemporal dementia, however, no evidence of the implication *APOE* genotype in ALS development risk has been found so far [740, 741].

Interestingly, alterations several receptors related to apoE have been related to the ALS phenotype. The study from Andres-Benito and colleagues revealed an altered gene expression of ABCA1 and ABC transporters in the cortex of ALS patients [742]. Genetic variants in the LXR are associated with age onset in ALS patients, being the LXR SNP rs2695121 variant associated with a 30% increase in ALS duration, but none of the genetic variants studied were linked to ALS risk [743].

Lipid dysregulation or lipid cacostasis is also one of the pathological hallmarks of ALS, contributing to neurodegeneration [744–746]. The study by Abdel-Khalik and colleagues reported a defective cholesterol metabolism in the brains of ALS patients [747]. Lipidomic analysis performed in the motor cortex and spinal cord of animal models of ALS revealed an abnormal lipid droplet accumulation in defective astrocytes and mitochondrial dysfunction [748].

The current advances in experimental techniques could be beneficial for future studies to determine the implications of *APOE* genotype in the lipid dysregulation mechanisms and the molecular pathways involved in ALS pathology.

### Multiple Sclerosis

MS is a neuroinflammatory and demyelinating relapsing–remitting disease that will course with selective cortical neuron damage [749]. The contribution of the *APOE* polymorphisms in MS onset has long been discussed, and often results have been inconsistent due to lack of power in the studies. For instance, *APOE4* is linked to a more rapid progression and severity of MS, however, the meta-analysis performed by Lill CM and colleagues in more than twenty-nine thousand subjects didn’t support a role of the *APOE4* and *APOE2* polymorphisms on MS susceptibility [750–752]. On the other hand, an extensive UK-Biobank study evidenced the potential health risks subjected to the expression of the different *APOE* alleles. The authors revealed an association between *APOE2* with an increased risk of suffering MS among other disease conditions, such as ischemic heart failure and hypercholesterolemia, which correlated with previous findings [753, 754]. This study also evidenced ethnic-derived physiological differences among *APOE4*-carriers, that should be considered for future studies [753].

Recent studies point to a lipid dysregulation in MS patients and cognitive dysfunction, in which LXRs gained attention as a potential target [755–757] since LXR activation ameliorated disease severity in experimental autoimmune encephalomyelitis (EAE) models [758, 759].

BBB dysregulation will also be one of the first cerebrovascular abnormalities observed during MS. A compromised BBB will allow the infiltration of T cells, B cells, and macrophages into the CNS, and has been associated with the development of cognitive impairment [760, 761]. *APOE* deficiency and *APOE4* overexpression in mice can accelerate BBB breakdown by activating the NF- κB and MMP9 pathway [376, 702]. Additionally, an evaluation of the impact of the *APOE4* genotype upon cognitive performance in early MS patients revealed that *APOE4* homozygosity was associated with lower cognitive performance, but this effect was absent in *APOE4* heterozygous or *APOE2* carriers [762, 763]

Glial cell activation is also known to contribute to the progression of MS. Schrirmer L. and colleagues reviewed glial cell diversity during MS, where they noted prior studies showing that White matter lesions during MS are more severe than those to gray matter and where astrocytes overexpress LRP1, and to phagocytose myelin at the injured sites [764–766]. Nonetheless, the possible interaction between these events and the *APOE* genotype is yet unknown.

Regarding the microglial response during MS, R. Plemel and colleagues studied microglial response after acute demyelination. The authors labeled microglia and CNS-associated macrophages to differentiate them from brain-infiltrating macrophages, and through single-cell RNA sequencing observed multiple microglial activation states, in which they found genes linked to DAM microglia, so as an *apoE* upregulation at the lesion sites [767]. Besides, activation of Trem2 in microglia increases the density of oligodendrocyte precursors in demyelinated areas, thus potentially promoting remyelination and axonal integrity [768].

Therefore, there are still many aspects of MS pathology in which the contribution of the *APOE* genotype could be further addressed, such as dyslipidemia related to MS and the possible implications of *APOE*, its isoforms, and its interacting receptors.

## Conclusions

The *APOE* genotype dramatically influences many aspects of cell function in the CNS and impacts the progression of AD and other CNS diseases characterized by overt inflammation. Remarkably, new *APOE* genetic variants are still being discovered and provide more insights into the impact of the *APOE* genotype in neurodegenerative diseases. For instance, the *APOE* Jacksonville mutation (V236E), characterized by a mutation in the lipid-binding region, was reported to reduce Aβ aggregation in 5xFAD mice and manifested the relevance of lipid metabolism during AD [78]. Further, the R251G variant evidenced by Rabinovici and colleagues induces a single amino acid switch at position 251 and has been initially associated with decreased risk of suffering AD [769].

Furthermore, the biological significance of altered apoE levels in the CNS or the periphery are still uncertain. For instance, low plasmatic levels of apoE are related to a higher risk of developing dementia [770]. However, it was recently reported that low presynaptic apoE levels could be related to AD resilience [463]. Thus, it would result in significant interest in future studies to determine the influence of the altered levels of apoE isoforms in different compartments as well as which cells acting as a source of apoE, neurons, astrocytes, microglia, pericytes, and the implications of each source.

The *APOE* genotype will also impact the microenvironment and functionality of the neighboring cells, and the molecular pathways triggered in consequence. Besides, additional genetic and external factors aside from apoE influencing neuroinflammation and neurodegeneration will also contribute to differences in the performance of memory tasks in young *versus* more age advanced stages. The latter statement refers to the study by Zolakei and colleagues in which they observed slower memory decay in middle-age *APOE4* carriers [771]. Additionally, it is relevant to highlight that up to date, it is still unclear how the total concentration of apoE nor its isoforms could be related to the Aβ, p-tau status, the degree of dementia, or affect other biological parameters.

Some points that could not be discussed further in depth in this review are the implications of peripheral *versus* CNS apoE and the impact of the gut-brain axis in the development of neurodegenerative diseases [772]. In line with this, it must also be noted that the *APOE* genotype can influence peripheral diseases such as cardiovascular diseases or macular degeneration. Paradoxically, the *APOE4* genotype protects against macular degeneration onset, whereas *APOE2* carriers presented an earlier disease onset [773]. The implications of microglial cells in degenerative eye diseases such as primary open-angle glaucoma and age-related macular degeneration have been previously described, and animal studies suggest targeting microglial cells as a potential treatment for eye disorders [774]. Ocular disorders present some common hallmarks of neurodegenerative diseases and CNS pathologies extensively discussed by other authors [775], such as the connection between apoE metabolism and retinal inflammation linked to age-related macular degeneration [776]. Therefore, the implications of the *APOE* genotype in inflammatory eye-related diseases and their connection to the development of Alzheimer’s disease are highly relevant.

So far, it is yet unknown to which extent the APOE alleles are expressed in heterozygotes; and whether the differences in the protein expression levels of each isoform could result from post-secretory or pre-secretory mechanisms influencing apoE levels, related, for instance, to the findings pointing to an inverse correlation found between plasma and CSF levels of apoE4/apoE3 in heterozygote individual studies [201].

The outcome of this extensive literature review supports a neurodegenerative disease-promoting role of apoE4, arguably through a toxic gain of function. Nevertheless, there is still much to investigate and learn from *APOE* physiology and its implication in the context of neurodegenerative diseases. Current therapeutic strategies include targeting *APOE4* as upstream as possible or its conversion to other isoforms, apoE lipidation, anti-apoE4 immunotherapies, antisense oligonucleotide therapies, among others including non-pharmacological approaches, such as diet changes and physical exercise. Techniques such as single-cell sequencing, novel approaches such as synTOF, female and male animal models of *APOE*, and clinical studies taking into account the *APOE* genotype will be of great value in puzzling out the role of *APOE* in neuroinflammation and neurodegeneration.

## Data Availability

Not applicable.

## Abbreviations

AAV: Adenoviral vectors
ABCA1: ATP binding cassette transporter A1
ABCG1: ATP Binding Cassette Subfamily G Member 1
AD: Alzheimer's disease
ADNI: Alzheimer’s Disease Neuroimaging Initiative
ALS: Amyotrophic Lateral Sclerosis
apoE: Apolipoprotein E protein
APOE: Human apolipoprotein E gene
ApoER2: Apolipoprotein E receptor 2
APP: Amyloid precursor protein
ARF6: ADP-ribosylation factor 6
ARIA: Amyloid-related imaging abnormalities
ARM: Activated-response microglia
ASOs: Antisense oligonucleotide
ATM: Axonal tract-associated microglia
Aβ: Amyloid beta
BBB: Blood–brain barrier
BDNF: Brain-derived neurotrophic factor
C1q: Molecule C1 from the complement system
CAA: Cerebral amyloid angiopathy
CLU/ApoJ: Clusterin
CNS: Central nervous system
CPM: Cycling/proliferating microglia
CSF: Cerebrospinal fluid
DAM: Disease-associated microglial phenotype
DAMPs: Danger-associated molecular patterns
e.g.: For example
EAE: Experimental autoimmune encephalomyelitis
EC: Entorhinal cortex
EOAD: Early-onset Alzheimer's disease
EVs: Extracellular vesicles
gal-3: Galectin-3
GWAS: Genome-wide association studies
HDL: High-density lipoproteins
HMGB1: High-mobility group box protein 1
HSPGs: Heparan sulfate proteoglycans
IDE: Insulin-degrading enzyme
IFN-γ: Interferon-γ
IL1-β: Interleukin 1-β
IRM: Injury-responsive microglia
ISF: Interstitial fluid
LBD: Lewy Body dementia
LBs: Lewy Bodies
LC: Locus coeruleus
LDAM: Lipid-droplet accumulating microglia
LDLR: Low-density lipoprotein receptor
LOAD: Late-onset Alzheimer's disease
LRP1: Low-density lipoprotein receptor-related protein 1
LRRs: Leucine-rich repeats
LTD: Long-term depression
LTP: Long-term potentiation
LXR: Liver X receptor
MAPK: Mitogen-activated protein kinase
*m-Apoe*: Mouse apolipoprotein E gene
MCI: Mild cognitive impairment
Mertk: C-Mer tyrosine kinase
MGnD: Microglia neurodegenerative phenotype
MSA: Multiple system atrophy
mTOR: Rapamycin
NAMPs: Neurodegeneration-associated molecular patterns
NFTs: Neurobibrillary tangles
NHE6: Sodium-hydrogen exchanger 6
NLRs: Inflammasome-forming nucleotide-binding oligomerization domain (nod)-like receptors
NSAIDs: Non-steroidal anti-inflammatory drugs
OPCs: Oligodendrocyte progenitor cells
PAMPs: Pathogen-associated molecular patterns
PD: Parkinson’s disease
PDD: Parkinson's disease with dementia
PET: Positron emission tomography
PI3K: Phosphatidylinositol 3-kinase
PiB: Pittsburgh compound B
PIP: Phosphoinositol phosphate
PIP2: Phosphoinositol biphosphate
PLCϒ2: Phospholipase CY2
Poly-P: Inorganic polyphosphate
PRRs: Pattern recognition receptors
PSEN1: Presenilin1
PSEN2: Presenilin 2
RAGE: Receptor for advanced glycation end-products
RXR: Retinoid X receptor
sAPPβ: Soluble APPβ
SNP: Single nucleotide polymorphism
SYK: Spleen tyrosine kinase
SYNJ1: Synaptojanin 1
SynTOF: Synaptometry time of flight
T2DM: Type 2 diabetes mellitus
TBI: Traumatic brain injury
TIR: Toll/IL-1 receptor
TLRs: Toll-like receptors
TNF-α: Tumor necrosis factor-α
TREM2: Receptor expressed on myeloid cells 2
TRM: Transiting-response microglia
tyrobp: Protein tyrosine kinase binding protein
Usp-18: Ubiquitin-specific protease 18
WAM: White-matter microglia
α-syn: Alpha-synuclein
